# Racial, Ethnic, Socioeconomic, and Geographic Inequities in Access to Mechanical Circulatory Support

**DOI:** 10.1016/j.jscai.2023.101193

**Published:** 2023-10-25

**Authors:** Ashwin S. Nathan, Kriyana P. Reddy, Lauren A. Eberly, Alexander Fanaroff, Howard M. Julien, Paul Fiorilli, Joyce Wald, Shafik Mutaawe, Marisa Cevasco, Christian Bermudez, Navin K. Kapur, Mir Babir Basir, Robert Roswell, Peter W. Groeneveld, Jay Giri

**Affiliations:** a Penn Cardiovascular Outcomes, Quality, and Evaluative Research Center, University of Pennsylvania, Philadelphia, Pennsylvania; b Division of Cardiology, Hospital of the University of Pennsylvania, Philadelphia, Pennsylvania; c Leonard Davis Institute of Health Economics, University of Pennsylvania, Philadelphia, Pennsylvania; d Corporal Michael J. Crescenz VA Medical Center, Philadelphia, Pennsylvania; e Department of Medicine, Perelman School of Medicine, University of Pennsylvania, Philadelphia, Pennsylvania; f Division of Cardiac Surgery, Hospital of the University of Pennsylvania, Philadelphia, Pennsylvania; g The CardioVascular Center, Tufts Medical Center, Boston, Massachusetts; h Henry Ford Hospital, Detroit, Michigan; i Zucker School of Medicine, Northwell Health, Hofstra University, Hempstead, New York

**Keywords:** access to care, inequities, temporary mechanical circulatory support

## Abstract

**Background:**

Hospital admissions for cardiogenic shock have increased in the United States. Temporary mechanical circulatory support (tMCS) can be used to acutely stabilize patients. We sought to evaluate the presence of racial, ethnic, and socioeconomic inequities in access to MCS in the United States among patients with cardiogenic shock.

**Methods:**

Medicare data were used to identify patients with cardiogenic shock admitted to hospitals with advanced tMCS (microaxial left ventricular assist device [mLVAD] or extracorporeal membranous oxygenation [ECMO]) capabilities within the 25 largest core-based statistical areas, all major metropolitan areas. We modeled the association between patient race, ethnicity, and socioeconomic status and use of mLVAD or ECMO.

**Results:**

After adjusting for age and clinical comorbidities, dual eligibility for Medicaid was associated with a 19.9% (95% CI, 11.5%-27.4%) decrease in odds of receiving mLVAD in a patient with cardiogenic shock (*P* < .001). After adjusting for age, clinical comorbidities, and dual eligibility for Medicaid, Black race was associated with 36.7% (95% CI, 28.4%-44.2%) lower odds of receiving mLVAD in a patient with cardiogenic shock. Dual eligibility for Medicaid was associated with a 62.0% (95% CI, 60.8%-63.1%) decrease in odds of receiving ECMO in a patient with cardiogenic shock (*P* < .001). Black race was associated with 36.0% (95% CI, 16.6%-50.9%) lower odds of receiving ECMO in a patient with cardiogenic shock, after adjusting for Medicaid eligibility.

**Conclusions:**

We identified large and significant racial, ethnic, and socioeconomic inequities in access to mLVAD and ECMO among patients presenting with cardiogenic shock to metropolitan hospitals with active advanced tMCS programs. These findings highlight systematic inequities in access to potentially lifesaving therapies.

## Highlights

• There exist metropolitan and nonmetropolitan disparities in access to advanced temporary mechanical circulatory support therapies.
• We identified racial and socioeconomic inequities in access to mechanical circulatory support.
• These findings highlight systemic inequities in the care of patients with cardiogenic shock.

## Central Illustration

**Figure:**
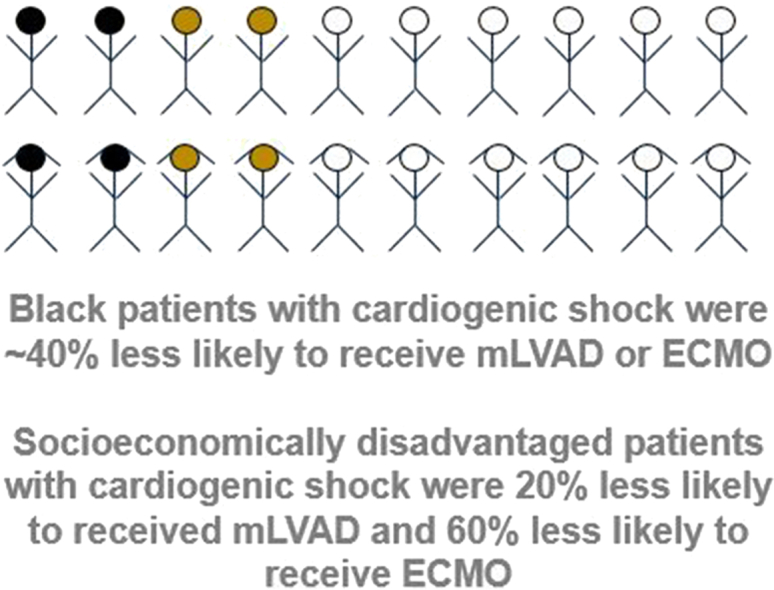

## Introduction

Hospital admissions for cardiogenic shock, characterized by severe myocardial dysfunction, impaired organ perfusion and high mortality, have increased in the United States over the last several decades.^1^^,^^2^ Acute pharmacologic therapies are limited to inotropic agents and vasoactive medications, which have limited benefits. Temporary mechanical circulatory support (tMCS) platforms provide increased and more reliable cardiac support when compared to pharmacologic therapies and most commonly include intra-aortic balloon pumps (IABPs), temporary microaxial left ventricular assist devices (mLVADs) and extracorporeal membranous oxygenation (ECMO).^3^ Over the last 2 decades, there has been an increase in the use of tMCS among patients with cardiogenic shock, though randomized data remain lacking.^4^

Although IABP therapy has been a long-standing and well-established therapeutic option, mLVAD and ECMO represent advanced, “high-technology” therapies that have increased in use in the modern era of shock management.^5^^,^^6^ Prior research has demonstrated substantial geographic as well as racial, ethnic, and socioeconomic inequities in access to “high-technology” procedures during the growth phase of novel cardiovascular technologies as they become established therapies.^7^, ^8^, ^9^

However, these prior studies evaluated elective procedures, whereas tMCS is implanted emergently to stabilize patients with cardiogenic shock who may otherwise rapidly deteriorate and die.^10^, ^11^, ^12^, ^13^ Inequities in timely access to tMCS may contribute to disparities in cardiogenic shock outcomes.^14^ Therefore, in this study, we sought to evaluate the presence of racial, ethnic, and socioeconomic inequities in access to tMCS and mLVAD in the United States.

## Methods

This study was deemed exempt by the institutional review board at the University of Pennsylvania. Research was carried out in accordance with the appropriate ethical guidelines per local institutional review board requirements.

### Study cohort

We used the Medicare Provider Analysis and Review and Master Beneficiary Summary data files to identify Medicare fee-for-service beneficiaries aged 66 years or older who were admitted to a percutaneous coronary intervention (PCI)-capable acute-care facility, had an admission or discharge diagnosis of cardiogenic shock, and underwent IABP, mLVAD, or ECMO between January 1, 2016, and December 31, 2019. Acute-care facilities were deemed PCI-capable if they coded for ≥10 PCI in a given year, which we chose to indicate the presence of a catheterization laboratory with interventional cardiologists who would have the capacity to obtain vascular access and insert mechanical circulatory support mechanical circulatory support (MCS). We further defined PCI-capable sites as primary PCI-capable (site coded for PCI for acute myocardial infarction), and/or elective PCI-capable (site coded PCI for acute myocardial infarction and for any other form of PCI). Among PCI-capable acute-care facilities, we determined sites that had cardiac surgery capabilities as those that coded for ≥10 cardiac surgery procedures in a given year. Cardiogenic shock was identified from any diagnosis code position using *International Classification of Diseases, Tenth Edition* (ICD-10) codes I50.21, I50.23, I50.41, I97.0, I97.110, I97.111, I97.130, I97.710, I97.711, I97.790, I97.791, I97.88, I97.89, and R57.0. IABP was identified with ICD-10 codes 5A02210 and 5A02110, mLVAD with codes 5A0221D and 5A0211D, and ECMO with code 5A15223.

All PCI-capable acute-care hospitals were considered candidate hospitals for the development of an mLVAD program as they are theoretically capable of performing high-risk and complex PCI with tMCS. All other hospitals were excluded from all analyses. Hospitals that performed ≥5 mLVAD, IABP, and/or ECMO procedures in a calendar year were defined as mLVAD, IABP, and/or ECMO programs, respectively, for that year and all subsequent years.

Patients and hospitals were assigned to individual core-based statistical areas (CBSAs) using CBSA to ZIP code crosswalks from the US Department of Housing as previously described.^7^^,^^8^^,^^15^^,^^16^ ZIP codes were classified as micropolitan (10,000-50,000 population) or metropolitan (≥50,000 population) based on the 2010 CBSA designation, and ZIP codes not linked to micropolitan or metropolitan CBSAs were designated as rural. Among metropolitan CBSAs with at least 1 mLVAD program, we identified the 25 largest metropolitan CBSAs by population per the 2010 US Census.

### Race, ethnicity, and socioeconomic identification

Medicare fee-for-service beneficiaries’ race and ethnicity were determined from Medicare Demographic Data files, and socioeconomic status was evaluated using 3 measures: median household income, dual-eligibility status for Medicaid, and the distressed communities index (DCI) score. Higher DCI values indicate higher community-level distress. Each marker was assessed at the level of ZIP code for patient residence.^17^

### Statistical analysis

First, we generated bar graphs to visualize PCI-capable acute-care hospitals offering IABP, mLVAD, and/or ECMO for each year of the study period. We then created separate bar graphs showing frequencies of sites offering IABP, mLVAD, and/or ECMO by year broken down by metropolitan designation, quartiles of median household income of patients served, quartiles of proportion of Black patients served, and quartiles of proportion of Hispanic patients served. Additionally, maps of US hospitals with and without mLVAD programs and hospitals with and without ECMO programs were generated.

Second, we compared characteristics of candidate hospitals with and without mLVAD programs using the *t* test for means and χ^2^ analysis for proportions, as appropriate. We further evaluated hospital characteristics in multivariable logistic regression models with the presence of an mLVAD program as the dependent variable. Covariates included hospital characteristics (number of beds, geographic region, for-profit status, teaching status, primary PCI capability, elective PCI capability, and cardiac surgery capability) and CBSA designation (metropolitan, micropolitan, or rural). As a sensitivity analysis, we conducted similar analyses to compare hospitals with and without ECMO programs.

Third, among candidate mLVAD hospitals, we identified indicators of socioeconomic status (median household income, percentage of Medicaid dual-eligible beneficiaries, and mean DCI) for all inpatients treated in the study period based on patient ZIP code information. We then employed *t* tests for means to compare the socioeconomic characteristics of patients served by hospitals with mLVAD programs and hospitals without mLVAD programs during the study period. We repeated these analyses for hospitals with and without ECMO programs during the study period.

Fourth, we evaluated the likelihood of receiving mLVAD among patients with cardiogenic shock at an mLVAD site in the 25 largest CBSAs with mLVAD programs. We generated generalized linear mixed effects models with binomial distribution and logit-link function to model the association between patient race, ethnicity, and socioeconomic status and insertion of mLVAD. This analysis was limited to the 25 largest CBSAs to assess mLVAD utilization against ZIP code-level socioeconomic characteristics and patient-level demographic characteristics in areas where geographic access to mLVAD is not a limitation. We clustered data at the hospital level using a mixed effects approach to better capture hospital-specific effects on mLVAD insertion. We adjusted for age, clinical comorbidities, race, and ethnicity. Each of the 3 socioeconomic indicators was introduced separately as a covariate into the model. These models were repeated separately with ECMO insertion as the dependent variable.

Fifth, we evaluated variation in ZIP-code level age-adjusted rates of mLVAD per 100,000 Medicare beneficiaries within the 25 largest CBSAs with mLVAD programs. We mapped choropleths of age-adjusted mLVAD rates by ZIP code alongside corresponding choropleths for ZIP-level proportions of Medicaid dual-eligible patients and proportions of Black or Hispanic patients. Choropleths were also generated for ZIP-level combined rates of mLVAD and ECMO.

Statistical analyses were performed using SAS version 9.4 (SAS Institute). Choropleths were generated using R version 4.1.2 (R Foundation for Statistical Computing). All statistical tests were predefined in an analytic plan and were 2-tailed, and *P* values <.05 were designated statistically significant. Normality was tested and met criteria for household income, dual eligibility for Medicaid, and DCI variables. There was minimal skew to the income data, but we felt the sample size was large enough to assume normality. Patients with missing data were excluded from the analysis; however, this was 1% or less of the cohort.

## Results

**Figure 1:**
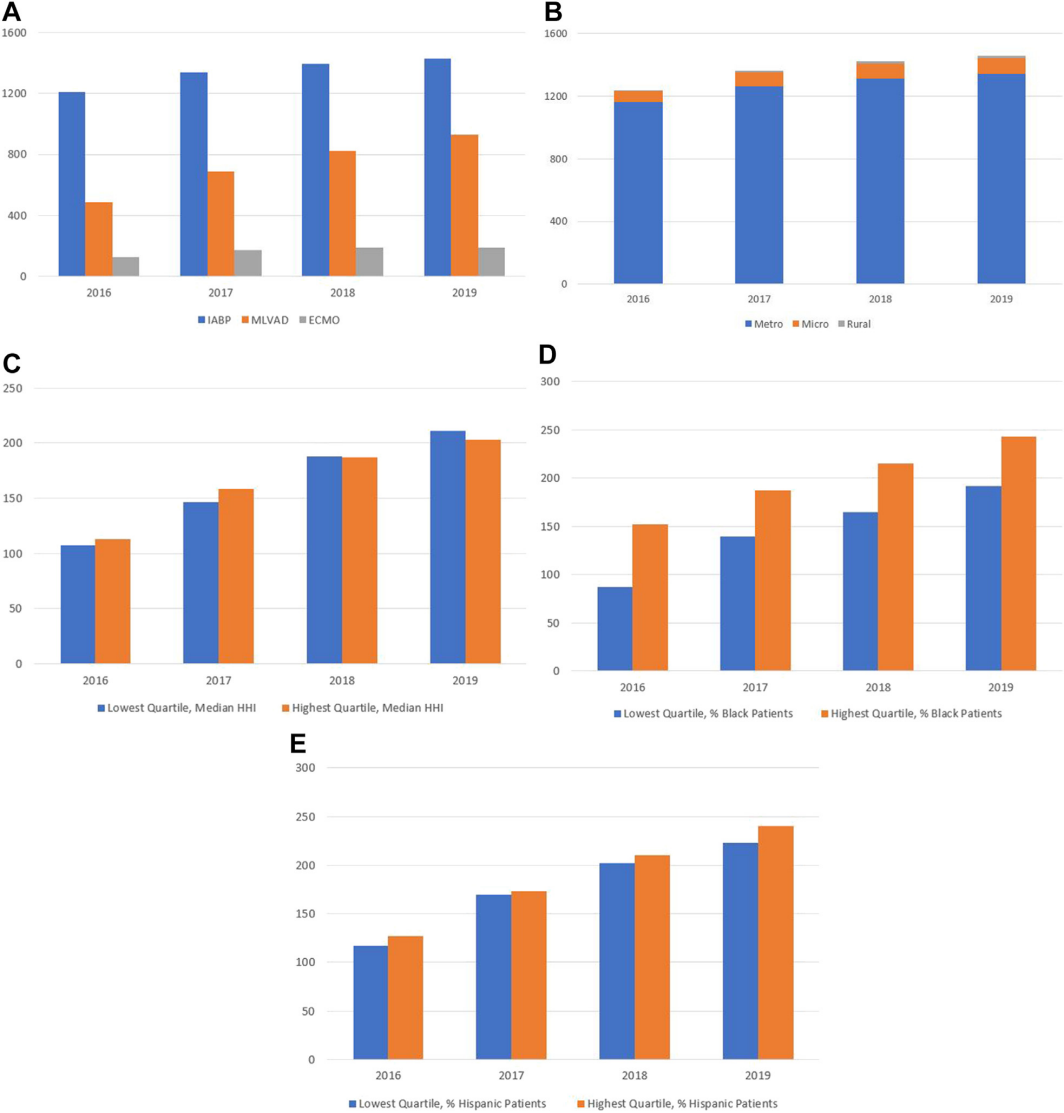
**Total acute-care hospitals with PCI capabilities offering mechanical LVAD, IABP, and/or ECMO for years 2016 to 2019.** (**A**) Total existing acute-care hospitals with PCI capabilities offering different MCS modalities. (**B**) Total acute-care hospitals with PCI capabilities offering MCS (at least 1 of IABP, mLVAD, or ECMO) by metropolitan designation. (**C**) Total acute-care hospitals with PCI capabilities offering advanced MCS (mLVAD or ECMO) serving patients in lowest and highest quartiles of median household income (HHI). (**D**) Total acute-care hospitals with PCI capabilities offering MCS (mLVAD or ECMO) in lowest and highest quartiles of proportion of Black patients served. (**E**) Total acute-care hospitals with PCI capabilities offering advanced MCS (mLVAD or ECMO) in lowest and highest quartiles of proportion of Hispanic patients served. ECMO, extracorporeal membranous oxygenation; IABP, intra-aortic balloon pump; MCS, mechanical circulatory support; mLVAD, microaxial left ventricular assist device; PCI, percutaneous coronary intervention.

In the Medicare Provider Analysis and Review data files, we identified 4780 inpatient prospective payment system hospitals and critical access hospitals in the 50 US states and the District of Columbia. Of those, 1829 were identified to be PCI-capable acute-care hospitals, which served as candidate hospitals in our analyses. In every year of the study period, most sites offering some form of tMCS, which is defined as offering at least one of IABP, mLVAD, or ECMO, were located in metropolitan areas. In 2019, 92% of tMCS-offering sites were in metropolitan areas, whereas 7% were located in micropolitan areas, and 1% were located in rural areas. Year over year, there were more hospitals offering advanced tMCS (mLVAD or ECMO) that served patients in the highest quartile of median household income than hospitals that served patients in the lowest quartile of median household income. There were also more hospitals offering advanced tMCS in the highest quartile of proportion of Black and Hispanic patients served than hospitals in the lowest quartile of proportion of Black and Hispanic patients served (Figure 1). Geographic distributions of candidate hospitals with and without mLVAD as well as candidate hospitals with and without ECMO are provided in Supplemental Figure S1.

We identified 929 hospitals that had established mLVAD programs by December 31, 2019. Hospitals with mLVAD programs tended to have ≥400 beds (*P* < .001) and be teaching hospitals (*P* < .001). Hospitals with mLVAD programs also were more likely to have cardiac surgery capability (*P* < .001) and elective PCI capability (*P* < .001) (Table 1). Cardiac surgery capability, elective PCI capability, nonprofit hospital status, and ≥400 beds were all associated with increased odds of hospitals having mLVAD programs in multivariable logistic regression analysis (Table 2). There were 191 hospitals with established ECMO programs. Hospitals with ECMO programs tended to have ≥400 beds (*P* < .001) and be teaching hospitals (*P* < .001) (Supplemental Table S1). Having ≥400 beds and teaching hospital status were associated with increased odds of hospitals having ECMO programs in multivariable logistic regression analysis (Supplemental Table S2).

**Table 1:** Characteristics of candidate hospitals with mLVAD programs compared with candidate hospitals without mLVAD programs.

Variable	No mLVAD (n = 900)	mLVAD (n = 929)	*P*
Bed size			<.001
<100 beds	151 (16.8)	29 (3.1)	
100-399 beds	689 (76.6)	548 (59)	
≥400 beds	60 (6.7)	352 (37.9)	
Teaching hospital	55 (6.1)	208 (22.4)	<.001
Profit status			.01
For-profit	198 (22)	179 (19.3)	
Nonprofit	581 (64.6)	656 (70.6)	
Government	121 (13.4)	94 (10.1)	
Region			.9
Midwest	224 (25.2)	224 (24.1)	
Northeast	124 (14)	131 (14.1)	
South	353 (39.8)	383 (41.2)	
West	187 (21.1)	191 (20.6)	
Geographic area			<.001
Metropolitan	733 (81.4)	884 (95.2)	
Micropolitan	148 (16.4)	42 (4.5)	
Rural	19 (2.1)	3 (0.3)	
Primary PCI capability	900 (100)	929 (100)	<.001
Elective PCI capability	889 (99)	929 (100)	<.001
Cardiac surgery capability	268 (29.8)	865 (93.1)	<.001

Values are presented as n (%).

mLVAD, microaxial left ventricular assist device; PCI, percutaneous coronary intervention.

**Table 2:** Association between the odds of hospitals having mLVAD programs and hospital factors among acute-care hospitals with PCI capability.

Variable	Odds ratio (95% CI)	*P*
Bed size (<100 beds as reference)		
100-399 beds	1.7 (1.2-2.4)	.001
≥400 beds	4.7 (3.1-6.9)	<.001
Teaching hospital (nonteaching as reference)	0.95 (0.72-1.3)	.7
CSBA categorization (rural as reference)		
Metropolitan	1.0 (0.37-2.6)	.97
Micropolitan	0.69 (0.25-1.9)	.47
Region (West as reference)		
Midwest	0.80 (0.65-0.99)	.04
Northeast	0.97 (0.74-1.3)	.84
South	1.2 (1.0-1.5)	.05
Profit status (government as reference)		
For profit	1.3 (0.9-1.7)	.13
Nonprofit	1.5 (1.1-1.9)	.003
PCI capability (primary as reference)		
Elective PCI capability	3.0 (1.2-7.6)	.02
Cardiac surgery capability	12.7 (10.6-15.2)	<.001

CSBA, core-based statistical areas; mLVAD, microaxial left ventricular assist device; PCI, percutaneous coronary intervention.

Hospitals with mLVAD programs treated patients with lower median household incomes (-$724; 95% CI, -$1344 to $103; *P* = .02) and fewer Medicaid dual-eligible patients (-1.0 percentage points; 95% CI, -1.5 to -0.6; *P* < .001) than hospitals without mLVAD programs (Table 3). Sensitivity analyses of hospitals with and without ECMO programs showed that hospitals with ECMO programs treated patients with higher median household incomes ($3205; 95% CI, $1889-$4521; *P* < .001), fewer Medicaid dual-eligible patients (-3.3; 95% CI, -5.0 to -1.7; *P* < .001), and patients from areas with lower DCI (-1.5; 95% CI, -2.5 to -0.5; *P* < .001) than hospitals without ECMO programs (Supplemental Table S3).

**Table 3:** Difference in socioeconomic characteristics of patients cared for by acute-care hospitals with PCI capabilities with and without mLVAD programs.

	mLVAD program (n = 929)	No mLVAD program (n = 900)	Difference (95% CI)	*P*
Median household income, $	55,060 ± 12,113	55,783 ± 14,738	-724 (-1344 to -103)	.02
Distressed communities index, unit	45.4 ± 15.0	45.4 ± 18.4	-0.02 (-0.8 to 0.75)	.95
Dual eligibility for Medicaid, %	13.4 ± 9.2	14.5 ± 10.6	-1.0 (-1.5 to -0.6)	<.001

Values are presented as mean ± SD.

mLVAD, microaxial left ventricular assist device; PCI, percutaneous coronary intervention.

In the 25 largest CBSAs, there were 284,277 patients with cardiogenic shock admitted to a mLVAD site during the study period. Of those patients, 5077 (1.8%) received mLVAD. Patients receiving a mLVAD had a mean (SD) age of 75.9 (7.0) years. Among patients receiving a mLVAD, 9.6% were Black, 5.0% were Asian, and 10.7% were Hispanic. The median (IQR) household income of patients receiving mLVAD was $61,419 ($45,826-$81,345), whereas the median (IQR) household income of patients not receiving mLVAD was $60,230 ($45,417-$80,339). Of patients receiving an mLVAD, 15.3% were Medicaid dual-eligible, and of patients not receiving an mLVAD, 20.4% were Medicaid dual-eligible. The median (IQR) community-level DCI score was 29.7 (12.4-56.0) among patients receiving an mLVAD, whereas the median (IQR) DCI score was 30.6 (13.4-57.7) among patients not receiving an mLVAD (Table 4).

**Table 4:** Baseline characteristics of Medicare fee-for-service beneficiaries aged 66 years and older with cardiogenic shock in the 25 largest CBSAs with mLVAD programs.

	Total	Receiving mLVAD	Not receiving mLVAD
Patients with cardiogenic shock, n	284,277	5077	279,200
Age, y	79.4 ± 8.5	75.9 ± 7	79.4 ± 8.5
Male sex	57.6	66.2	55.7
Race and ethnicity			
White	71.5	71.9	71.5
Black	13.7	9.6	13.7
Asian	3.8	5	3.8
Hispanic	8.9	10.7	8.8
Comorbidities			
Congestive heart failure	62.1	38.2	62.5
Hypertension	80.7	68.6	80.9
Diabetes	47.9	51.4	47.9
Stroke	6.8	5.8	6.9
Peripheral vascular disease	29.5	28.7	29.5
Kidney disease	56.7	49.5	56.8
Liver disease	7.8	6.3	7.8
Region			
Midwest	21.2	18.7	21.3
Northeast	27.4	26.5	27.4
South	28.4	28.1	28.4
West	23	26.8	22.9
Elixhauser comorbidities, n	7 (4-10)	5 (3-8)	7 (4-10)
Weighted AHRQ comorbidity score	22 (11-32)	18 (10-28)	22 (11-32)
Proportion of patients dually eligible for Medicaid	20.3	15.3	20.4
Household income, $	60,230 (45,467-80,339)	61,419 (45,826-81,345)	60,230 (45,417-80,339)
Distressed communities index score	30.6 (13.3-57.7)	29.7 (12.4-56.0)	30.6 (13.4-57.7)

Vales are %, mean ± SD, or median (IQR).

AHRQ, Agency for Healthcare Research and Quality; CBSA, core-based statistical area; mLVAD, microaxial left ventricular assist device.

The results of our generalized linear mixed effects models identifying associations between socioeconomic status, race, ethnicity, and likelihood of mLVAD insertion among patients with cardiogenic shock at mLVAD sites in the 25 largest CBSAs are presented in Table 5. For each $1000 decrease in median household income, the odds of receiving mLVAD for a patient with cardiogenic shock were 0.21% (95% CI, 0.08%-0.34%) lower (*P* = .002). Dual eligibility for Medicaid was associated with a 19.9% (95% CI, 11.5%-27.4%) decrease in odds of receiving mLVAD in a patient with cardiogenic shock (*P* < .001). After adjusting for median household income, Black race was associated with 36.7% (95% CI, 28.2%-44.1%) lower odds of receiving mLVAD in a patient with cardiogenic shock (*P* < .001). Similarly, after adjusting for dual eligibility for Medicaid, Black race was associated with 36.7% (95% CI, 28.4%-44.2%) lower odds of receiving mLVAD in a patient with cardiogenic shock. The results of our generalized linear mixed effects models identifying associations between socioeconomic status, race, ethnicity, and likelihood of ECMO insertion among patients with cardiogenic shock at ECMO sites in the 25 largest CBSAs are presented in Supplemental Table S4. Larger effect sizes were noted in the sensitivity analysis modeling odds of ECMO insertion among patients with cardiogenic shock in the 25 largest CBSAs with ECMO programs. For each $1000 decrease in median household income, the odds of receiving ECMO for a patient with cardiogenic shock were 0.65% (95% CI, 0.38%-0.93%) lower (*P* < .001). Dual eligibility for Medicaid was associated with a 62.0% (95% CI, 60.8%-63.1%) decrease in odds of receiving ECMO in a patient with cardiogenic shock (*P* < .001). Black race was associated with a 36.0% (95% CI, 16.6%-50.9%) lower odds of receiving ECMO in a patient with cardiogenic shock, after adjusting for Medicaid eligibility (Supplemental Table S5).

**Table 5:** Association between socioeconomic status, race, ethnicity, and likelihood of receiving mLVAD among patients with cardiogenic shock at an mLVAD hospital in the 25 largest CBSAs with mLVAD programs.

	Difference in odds of receiving mLVAD, %	*P*
Median household income (per $1000 decrease)	-0.21 (-0.34 to -0.08)	.002
Black race (binary)	-36.7 (-44.1 to -28.2)	<.001
Hispanic ethnicity (binary)	0.74 (-10.7 to 13.6)	.25
Dual eligibility for Medicaid (binary)	-19.9 (-27.4 to -11.5)	<.001
Black race (binary)	-36.7 (-44.2 to -28.4)	<.001
Hispanic ethnicity (binary)	11.3 (-1.9 to 26.3)	.10
Distressed communities (per 1-unit increase)	-0.22 (-0.36 to -0.07)	.003
Black race (binary)	-36.1 (-43.8 to -27.3)	<.001
Hispanic ethnicity (binary)	9.1 (-3.9 to 23.9)	.18

Adjusted for sex, age, and clinical comorbidities.

CBSA, core-based statistical area; mLVAD, microaxial left ventricular assist device.

Choropleths of the studied metropolitan CBSAs showed that ZIP codes having higher age-adjusted rates of mLVAD per 100,000 Medicare beneficiaries had smaller proportions of Medicaid dual-eligible patients and lower proportions of Black and/or Hispanic patients (Supplemental Figure S2). Choropleths of combined mLVAD and ECMO rates per 100,000 Medicare beneficiaries showed similar results and are presented in Supplemental Figure S3.

## Discussion

In an analysis of Medicare beneficiaries to understand the use of tMCS in the United States, we found an increase in mLVAD and ECMO programs with a concentration of advanced tMCS programs within metropolitan areas. tMCS-capable hospitals that offered mLVAD or ECMO treated fewer Medicaid-eligible patients when compared with hospitals that did not offer mLVAD or ECMO. Among patients admitted with cardiogenic shock to advanced tMCS programs within major metropolitan areas, socioeconomic distress was associated with significantly lower mLVAD and ECMO use. Further, after adjusting for socioeconomic status, Black race was associated with significantly lower mLVAD and ECMO use (Central Illustration). These findings highlight marked inequities in access to a potentially acute lifesaving therapy.

**Central Illustration:**
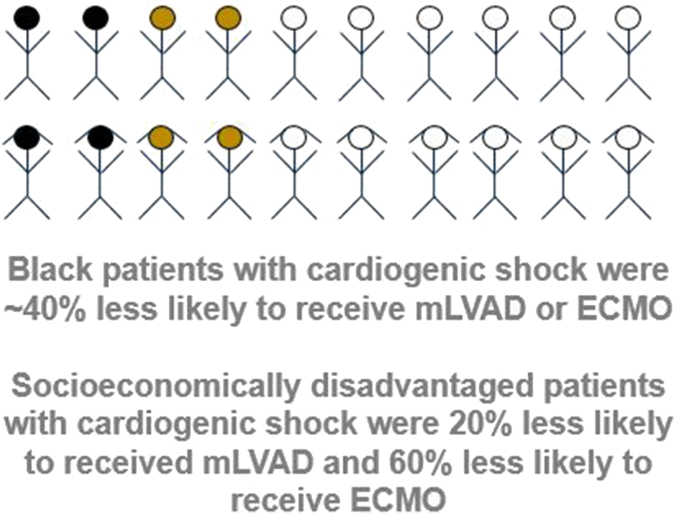
Measured inequities in access to mechanical circulatory support. ECMO, extracorporeal membranous oxygenation; mLVAD, microaxial left ventricular assist device.

The growth of tMCS programs has been substantial in the last several years, though concentrated within metropolitan areas. Between 2016 and 2019, while the numbers of IABP and ECMO programs remained relatively stable, there was almost a doubling of mLVAD programs in the United States. Although the presence of cardiac surgery was the strongest predictor of establishing an mLVAD program, almost 10% of hospitals without mLVAD programs did not have cardiac surgery capabilities. In addition, nearly 30% of hospitals with cardiac surgery capabilities did not establish a mLVAD program during the study period. There were only 45 PCI-capable, acute-care hospitals in the country in a nonmetropolitan area that offered mLVAD and only 3 within rural areas. Among hospitals offering ECMO, there was only 1 in the country within a rural area.

These findings represent a dramatic and dangerous limitation in access to advanced tMCS therapies for cardiogenic shock patients presenting to nonmetropolitan hospitals. Differential access to these platforms, despite a lack of clear randomized data, nonetheless represents an inequity in care for rural patients. Cardiogenic shock may progress unpredictably, necessitating urgent decisions regarding its management, including the use of salvage tMCS to stabilize a deteriorating patient. Limited access to advanced tMCS services can therefore be fatal for patients presenting with shock to nonmetropolitan hospitals, as the patient can deteriorate during an attempted transfer to a center offering advanced tMCS.^11^^,^^18^ Although training and credentialing of physicians within nonmetropolitan hospitals may increase use, consideration should be given toward urgent mobilization of physicians from metropolitan areas to nonmetropolitan hospitals to assist locally. There have been examples of highly successful initiatives to improve access to ECMO using mobile physician and perfusion teams.^19^ Similar teams that may be able to assist with mLVAD use could improve the management of patients presenting with cardiogenic shock to nonmetropolitan hospitals.

Hospitals with advanced tMCS programs were concentrated within metropolitan areas and took care of overall patient populations that had significant Black, Hispanic, and socioeconomically disadvantaged patient populations, likely reflecting the urban location of these hospitals. Despite this, among Medicare beneficiaries with cardiogenic shock admitted in acute-care hospitals in metropolitan areas with active mLVAD programs, poorer patients were significantly less likely to receive mLVAD implantation after adjusting for clinical comorbidities. For each $50,000 decrease in median household income, there was a 10% associated reduced odds of receiving mLVAD. Dual-eligible patients for Medicaid with cardiogenic shock were 20% less likely to receive mLVAD implantation. Moreover, after adjusting for each of the socioeconomic variables, Black patients with cardiogenic shock had an almost 40% reduction in the odds of receiving mLVAD therapy. Even larger effect sizes were seen with ECMO use, with a 60% reduction in the odds of ECMO utilization among Medicaid dual-eligible patients. Black patients with cardiogenic shock had a similar 30% to 40% reduction in the odds of receiving ECMO insertion. We did not observe significant reductions in utilization of tMCS among Hispanic patients in unadjusted or adjusted analyses and Asian patients in unadjusted analyses.

Our group and others have highlighted inequities in access to advanced, “high-technology” cardiovascular therapies among patients cared for in areas and hospitals with access to these procedures.^7^, ^8^, ^9^ Although mLVAD and ECMO also represent “high-technology” therapies with growing use nationally that suffer similar rural/urban divides, they are distinct from elective procedures such as transcatheter aortic valve replacement or left atrial appendage occlusion in that they represent acute interventional therapies that are necessarily implanted during an inpatient admission. For a patient with cardiogenic shock, the decision to utilize advanced tMCS is urgent, if not emergent. As such, the mechanism of inequities that we found may be distinct from elective procedures such as transcatheter aortic valve replacement and left atrial appendage occlusion and are unlikely to represent issues in the outpatient treatment and referral pathway, as in those procedures, but rather to reflect the presence of significant unconscious biases and systemic racism within the inpatient acute-care health care system and the allocation of durable therapies such as left ventricular assist device (LVAD) and orthotopic heart transplantation (OHT).

mLVAD and ECMO are used as bridging therapies to recovery for acute cardiogenic shock but also as a bridge to more durable therapy for cardiogenic shock and advanced heart failure such as LVAD implantation and OHT. Several analyses have demonstrated significant inequities in access to LVAD and OHT among racially and ethnically minoritized patient populations.^20^^,^^21^ Limitation in the use of advanced tMCS therapies may stem from biases in decision making and systemic racism whereby physicians reduce allocating durable advanced heart failure therapies for minoritized groups of patients with cardiogenic shock.^22^ As such, marginalized patients who are denied durable long-term options of LVAD implantation or OHT due to unconscious bias or systemic racism may be pre-emptively denied bridging advanced tMCS therapies if they are felt to be a “bridge to nowhere.”

Taken together, the findings of this study highlight metropolitan and nonmetropolitan inequities in access to advanced tMCS therapies but also racial, ethnic, and socioeconomic inequities in access to mLVAD and ECMO among patients presenting with cardiogenic shock to metropolitan hospitals with active tMCS programs. Though there are not randomized data to justify routine mLVAD or ECMO use in cardiogenic shock patients, the differences in utilization among different racial, ethnic, and socioeconomic subgroups of patients with cardiogenic shock remain striking, nonetheless. These data follow an unfortunate, repetitive pattern of systemic racism present in medicine, though at a scale larger than previous observations, with an urgent need to address and impact these issues with systematic approaches to health equity.^23^^,^^24^

There are several limitations of the study. First, our study of patient-level access is limited to Medicare beneficiaries. This represents an over 65 years of age population for whom OHT use may be limited, though LVAD use may be an option. Nonetheless, it is important to note that advanced tMCS can be utilized as a bridge to recovery even among patients not eligible for advanced heart failure care. Second, our study is limited to the use of administrative claims data. The classification and urgency of cardiogenic shock is broad and requires the integration of multiple clinical variables that are not well captured with administrative data. Moreover, we are unable to assess for prohibitive patient-level factors such as occlusive iliofemoral arterial disease, aortic regurgitation, active hemorrhage, or other clinical drivers of decision making in tMCS utilization. However, the use of a national administrative database allows us to assess national and geographic trends in use. Third, 2 of the socioeconomic markers (median household income and DCI) are derived from ZIP code level data from the patients’ home addresses and are subject to ecological fallacy. However, dual eligibility for Medicaid services is determined at the individual patient level and had consistent results with the other 2 measures of socioeconomic distress. Finally, this study included only Medicare beneficiaries with a diagnosis of cardiogenic shock. We did not study the overall use of tMCS therapies in all scenarios in which it could be used (eg, supported, high-risk PCI, undifferentiated shock, postcardiac surgery). Less than 0.6% of the study population received any form of tMCS, which limits the generalizability of the findings; however, the stark relative differences in utilization between marginalized and nonmarginalized groups highlight significant inequities.

## Conclusion

In a national study using Medicare administrative data, there exist metropolitan and nonmetropolitan disparities in access to advanced tMCS therapies. Furthermore, we identified racial and socioeconomic inequities in access to mLVAD and ECMO among patients presenting with cardiogenic shock to metropolitan hospitals with active tMCS programs. These findings highlight both geographic inequities in access to tMCS but also within geographic areas that have ready availability of these therapies, racial and socioeconomic inequities in access to these procedures.
