# Supplementary material for: Racial, Ethnic, Socioeconomic, and Geographic Inequities in Access to Mechanical Circulatory Support
Source: J Soc Cardiovasc Angiogr Interv. 2023 Oct 25;3(1):101193. doi: 10.1016/j.jscai.2023.101193 (PMC11307759; doi:10.1016/j.jscai.2023.101193)

**Supplemental Tables & Figures**

**Supplemental Figure 1**.

PCI-capable acute care hospitals with MLVAD and ECMO programs as of December 31, 2019.


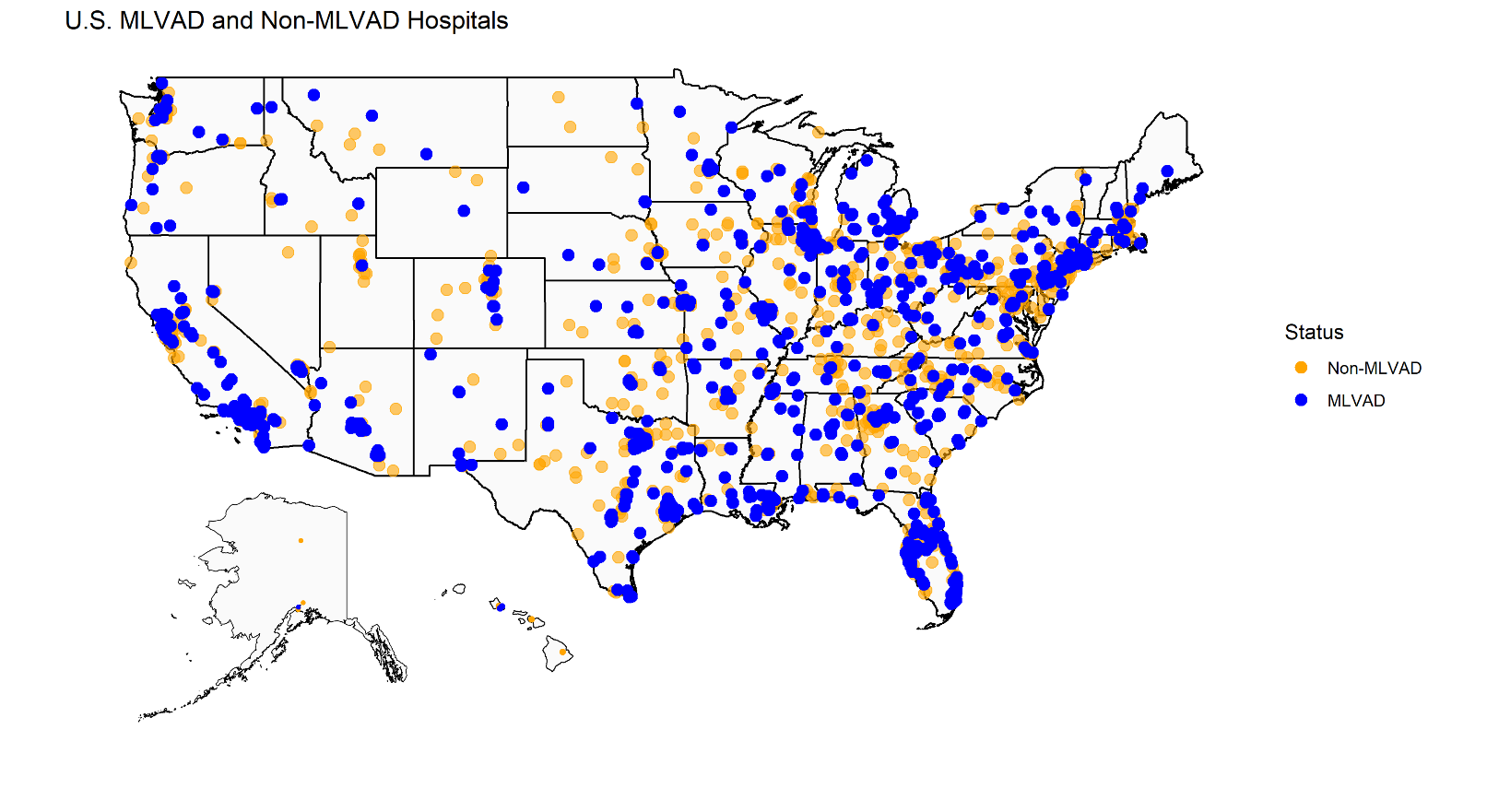

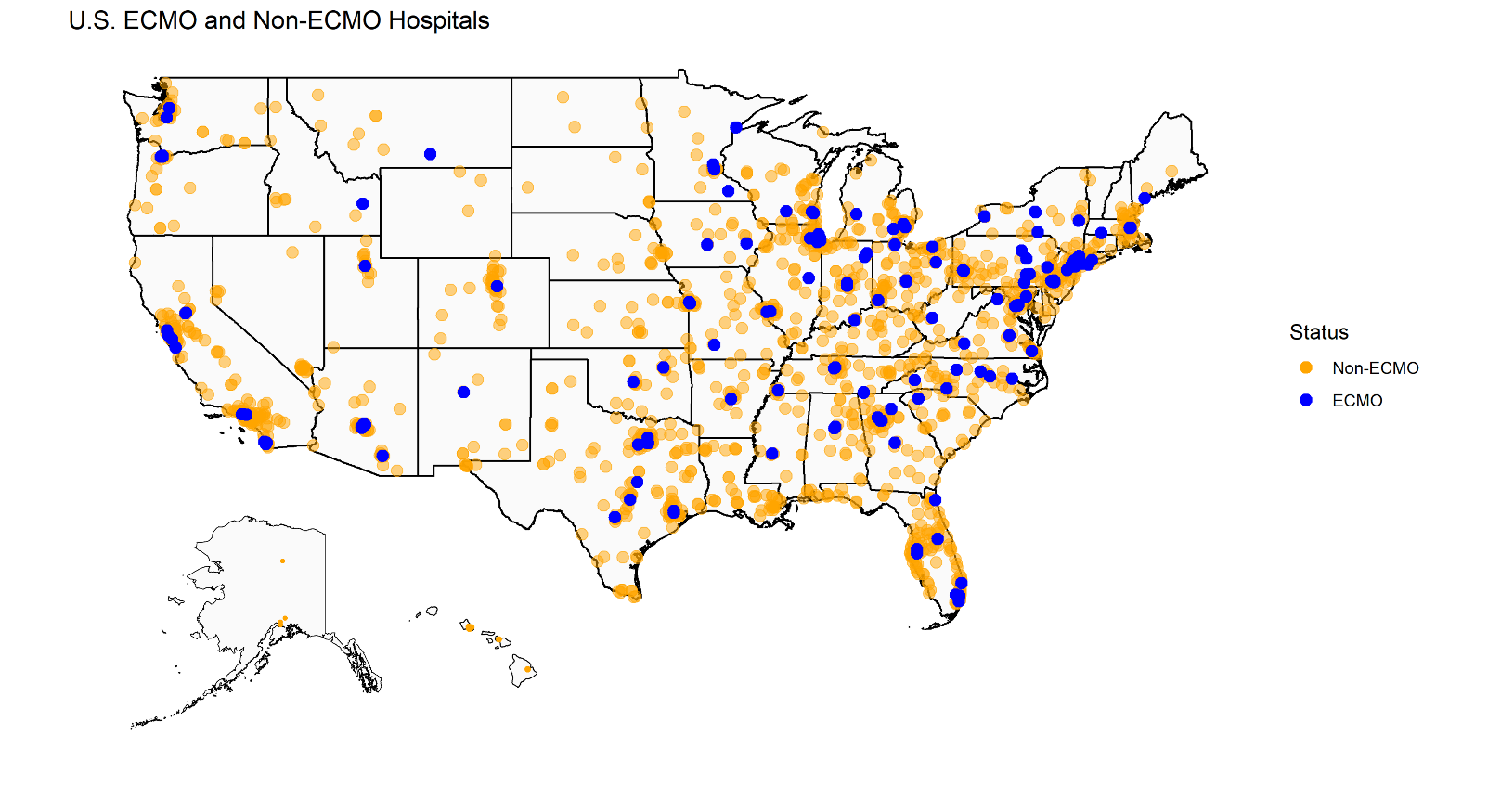


**Supplemental Table 1**. Characteristics of candidate hospitals with ECMO programs compared with candidate hospitals without ECMO programs.

| **Variable** | **No ECMO (n=1638)** | **ECMO (n=191)** | **P-Value** |
| --- | --- | --- | --- |
| Bed size, n (%) |  |  | <0.001 |
| < 100 Beds | 180 (11) | 0 (0) |  |
| 100-399 Beds | 1206 (74) | 31 (16) |  |
| ≥ 400 Beds | 252 (15) | 160 (84) |  |
| Teaching Hospital, n (%) | 134 (8.2) | 129 (67.5) | <0.001 |
| Profit status, n (%) |  |  | <0.001 |
| For-profit | 367 (22.4) | 10 (5.2) |  |
| Non-profit | 1087 (66.4) | 150 (78.5) |  |
| Government | 184 (11.2) | 31 (16.2) |  |
| Region, n (%) |  |  | <0.001 |
| Midwest | 404 (24.8) | 44 (23.2) |  |
| Northeast | 207 (12.7) | 48 (25.3) |  |
| South | 668 (41.1) | 68 (35.8) |  |
| West | 348 (21.4) | 30 (15.8) |  |
| Geographic Area, n (%) |  |  | <0.001 |
| Metropolitan | 1427 (87.1) | 190 (99.5) |  |
| Micropolitan | 190 (11.6) | 0 (0) |  |
| Rural | 21 (1.3) | 1 (0.5) |  |
| Primary PCI Capability, n (%) | 1638 (100) | 191 (100) | <0.001 |
| Elective PCI Capability, n (%) | 1627 (99) | 191 (100) | 0.26 |
| Cardiac Surgery Capability, n (%) | 943 (57.6) | 190 (99.5) | <0.001 |

**Supplemental Table 2**. Association between the Odds of Hospitals Having ECMO Programs and Hospital Factors Among Acute-Care Hospitals with PCI Capability

| **Variable** | **OR (95% CI)** | **P-Value** |
| --- | --- | --- |
| Bed Size (<100 beds as reference) |  |  |
| 100-399 beds | 9.6E8 (6.6E8 to 1.4E8) | <0.001 |
| >= 400 beds | 7.2E8 (7.2E8 to 7.2E8) | <0.001 |
| Teaching hospital (nonteaching as reference) | 4.4 (3.2 to 6.2) | <0.001 |
| CSBA Categorization (Rural as reference) |  |  |
| Metropolitan | NA | NA |
| Micropolitan | NA | NA |
| Region (West as reference) |  |  |
| Midwest | 0.85 (0.53 to 1.4) | 0.48 |
| Northeast | 1.2 (0.7 to 1.9) | 0.59 |
| South | 1.0 (0.65 to 1.6) | 0.95 |
| Profit status (Government as reference) |  |  |
| For profit | 0.79 (0.41 to 1.5) | 0.49 |
| Nonprofit | 1.4 (0.87 to 2.1) | 0.18 |
| PCI Capability (Primary as reference) |  |  |
| Elective PCI Capability | NA | NA |
| Cardiac Surgery Capability | NA | NA |

**Supplemental Table 3.** Difference in Socioeconomic Characteristics of Patients Cared for by acute care hospitals With and Without ECMO Programs

|  | **ECMO Program (n=191)** | **No ECMO Program (n=1638)** | **Difference (95% CI)** | **P Value** |
| --- | --- | --- | --- | --- |
| Median Household Income ($), Mean (SD) | 58,429 (11,988) | 55,224 (13,526) | 3,205 (1,889 to 4,521) | <0.001 |
| Distressed Communities Index (unit), Mean (SD) | 42.2 (12.2) | 45.6 (17.0) | -3.3 (-5.0 to -1.7) | <0.001 |
| Dual Eligibility for Medicaid (%), Mean (SD) | 12.5 (7.7) | 14.0 (10.0) | -1.5 (-2.5 to -0.5) | <0.001 |

**Supplemental Table 4**. Association Between Socioeconomic Status, Race, Ethnicity and Likelihood of Receiving ECMO among patients with cardiogenic shock at an ECMO Hospital in the 25 largest CBSAs with ECMO Programs. Adjusted for sex, age, and clinical comorbidities.

|  | Difference in Odds of Receiving ECMO, % | P-Value |
| --- | --- | --- |
| Median Household Income (per $1000 decrease) | -0.65 (-0.93 to -0.38) | <0.001 |
| Black Race (binary) | -35.3 (-50.6 to -15.3) | 0.002 |
| Hispanic Ethnicity (binary) | -11.3 (-38.7 to 28.1) | 0.52 |
|  |  |  |
| Dual Eligibility for Medicaid (binary) | -62.0 (-63.1 to -60.8) | <0.001 |
| Black Race (binary) | -36.0 (-50.9 to -16.6) | 0.001 |
| Hispanic Ethnicity (binary) | 2.3 (-28.9 to 47.0) | 0.9 |
|  |  |  |
| Distressed Communities (per 1-unit increase) | -0.89 (-1.2 to -0.53) | <0.001 |
| Black Race (binary) | -30.7 (-47.7 to -8.2) | 0.01 |
| Hispanic Ethnicity (binary) | -8.4 (-37.2 to 33.6) | 0.65 |

**Supplemental Figure 2**. Age-adjusted mLVAD Rates among all Medicare beneficiaries, % Dual Eligibility for Medicaid, and % Black or Hispanic patients in ZIP Codes in 25 largest CBSAs with mLVAD programs.


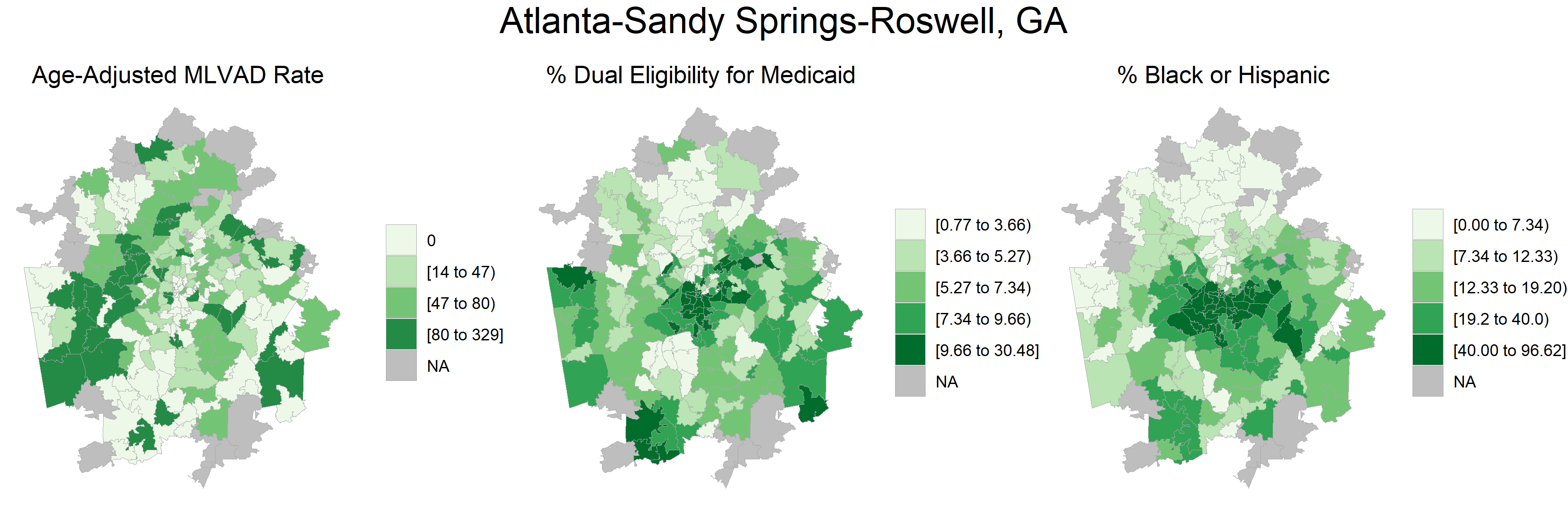

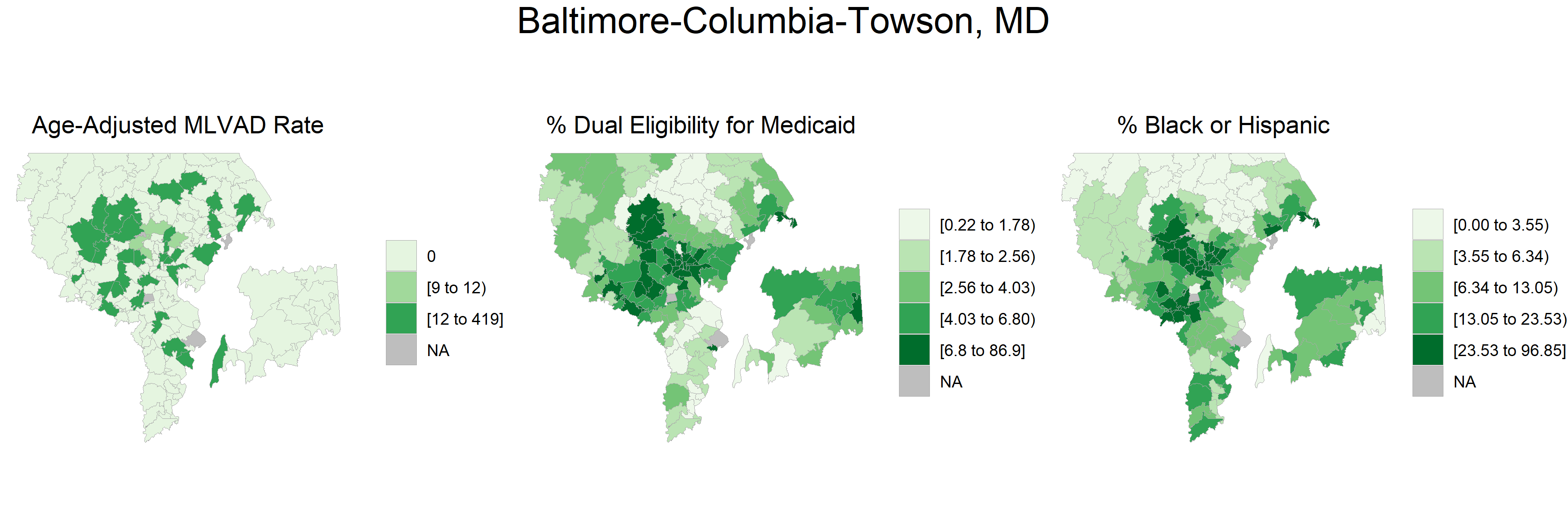

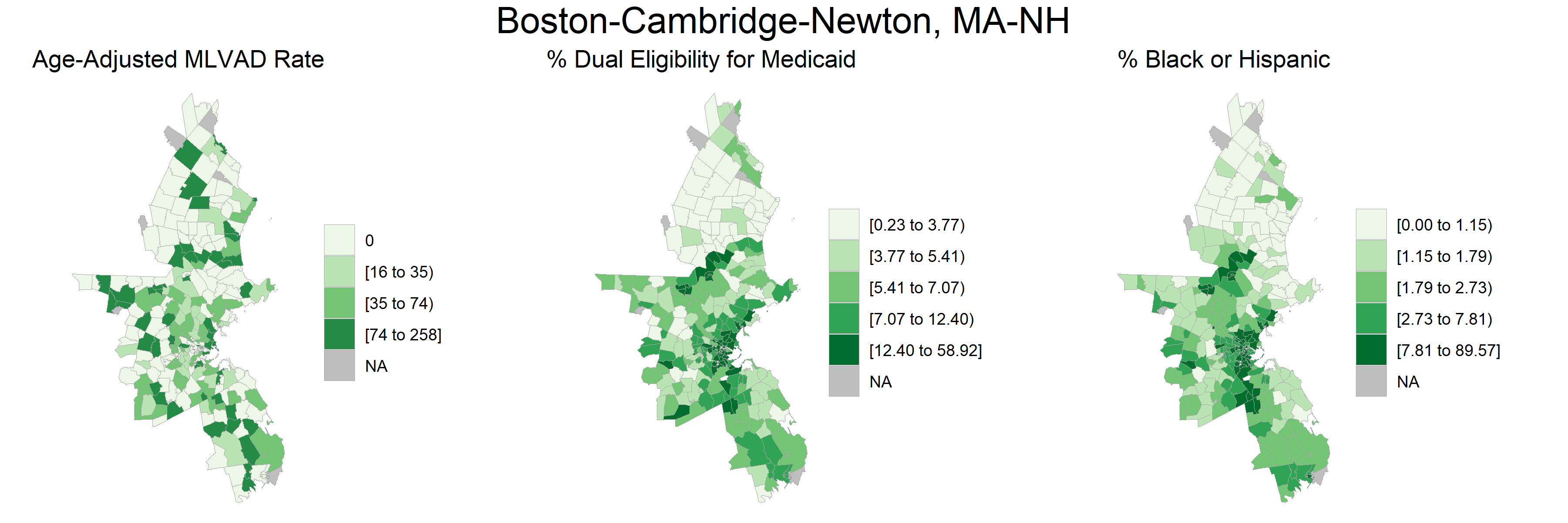

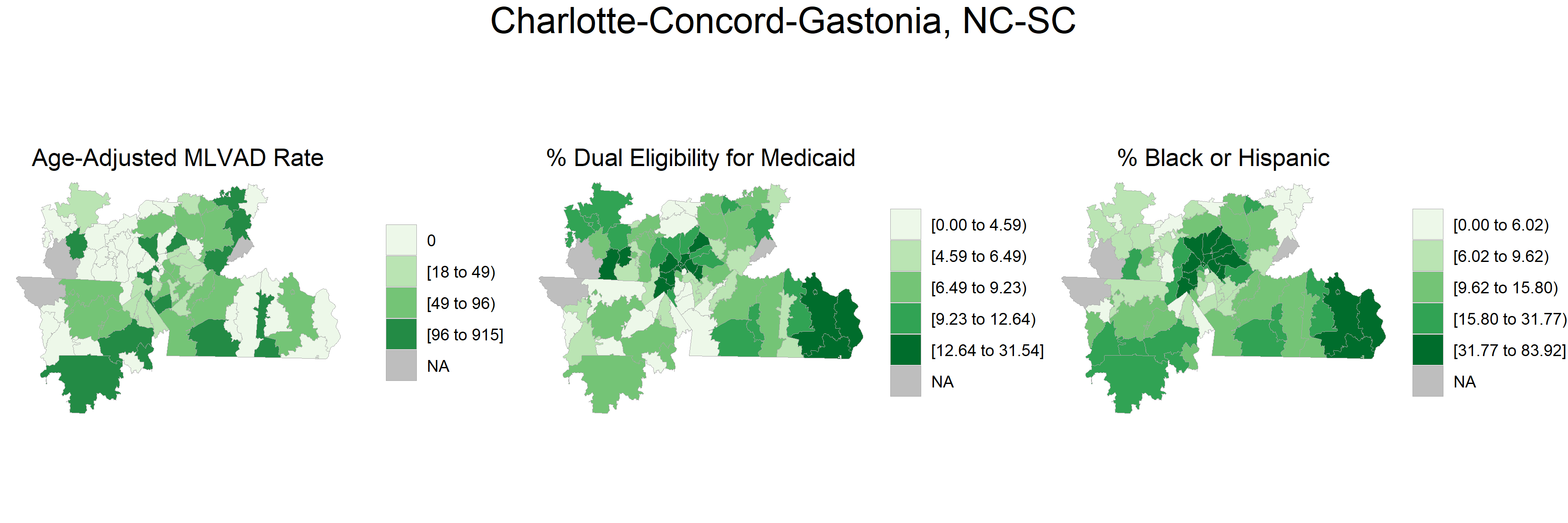

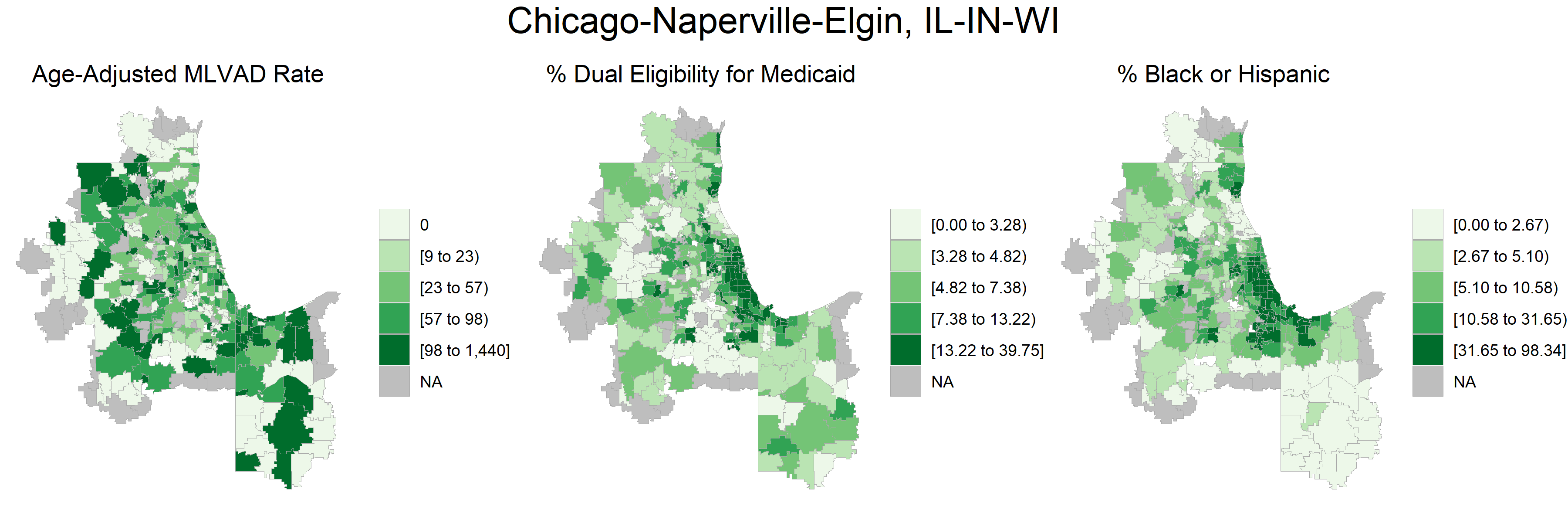

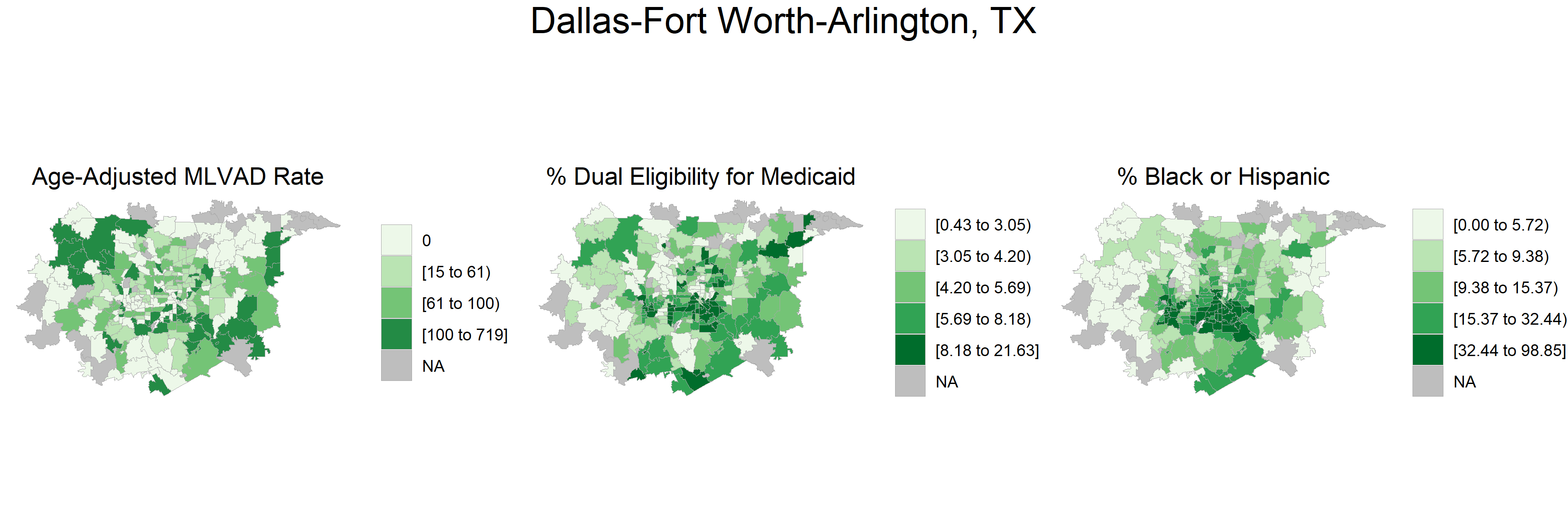

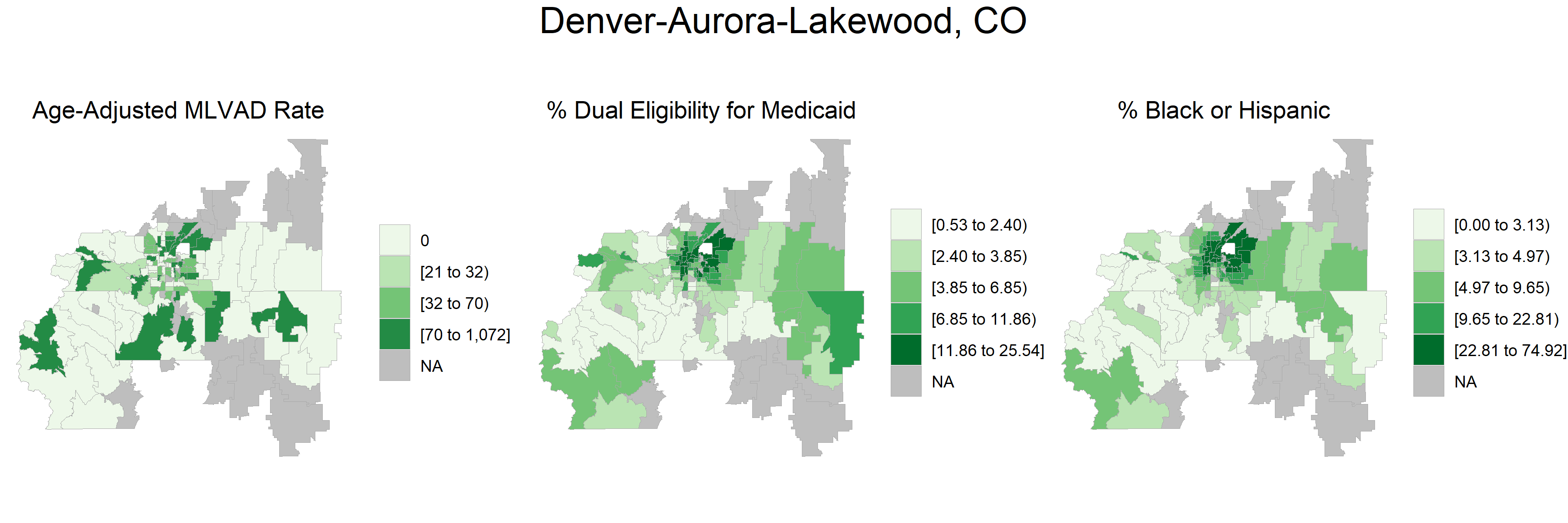

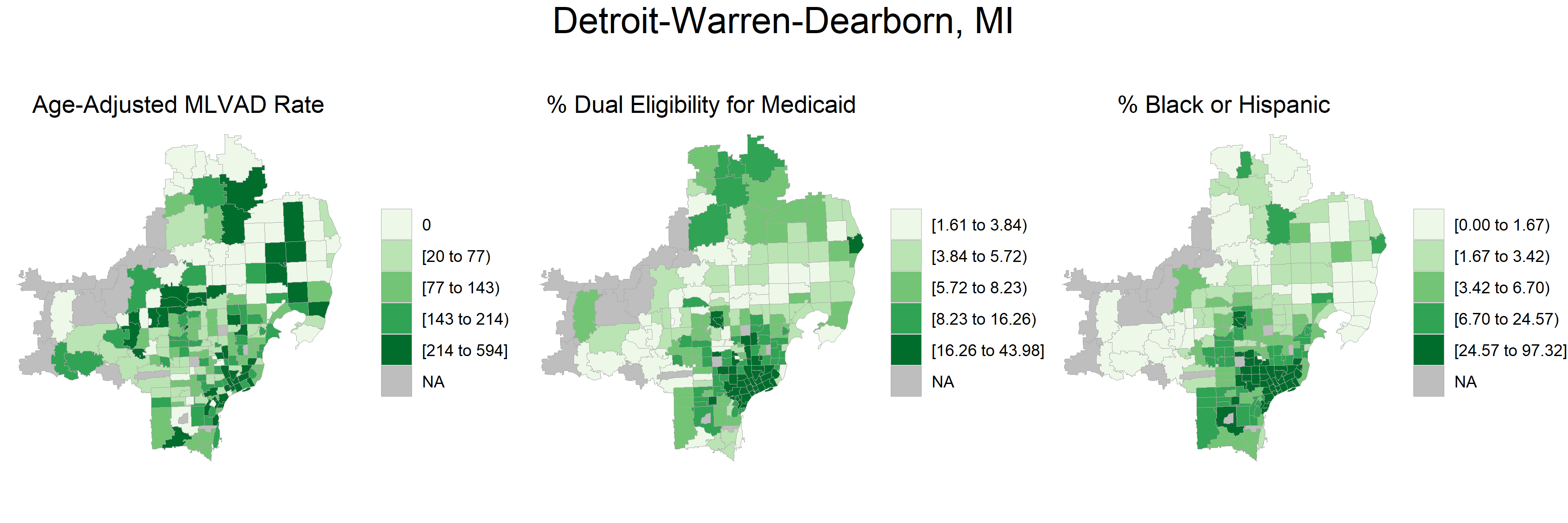

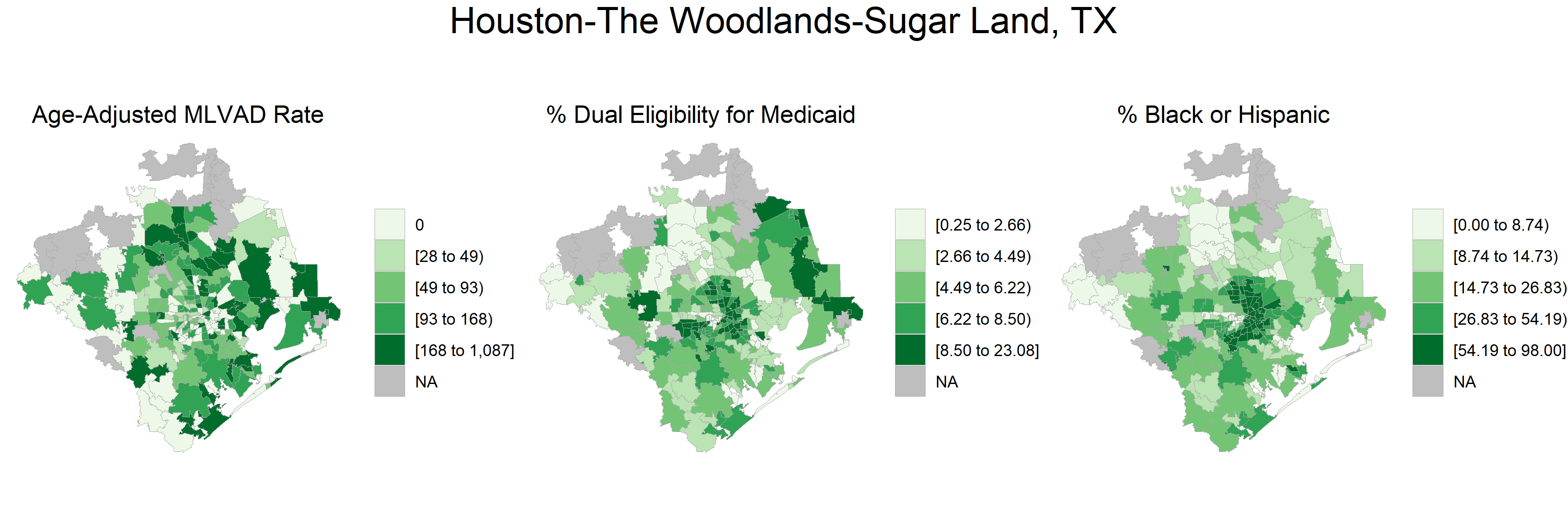

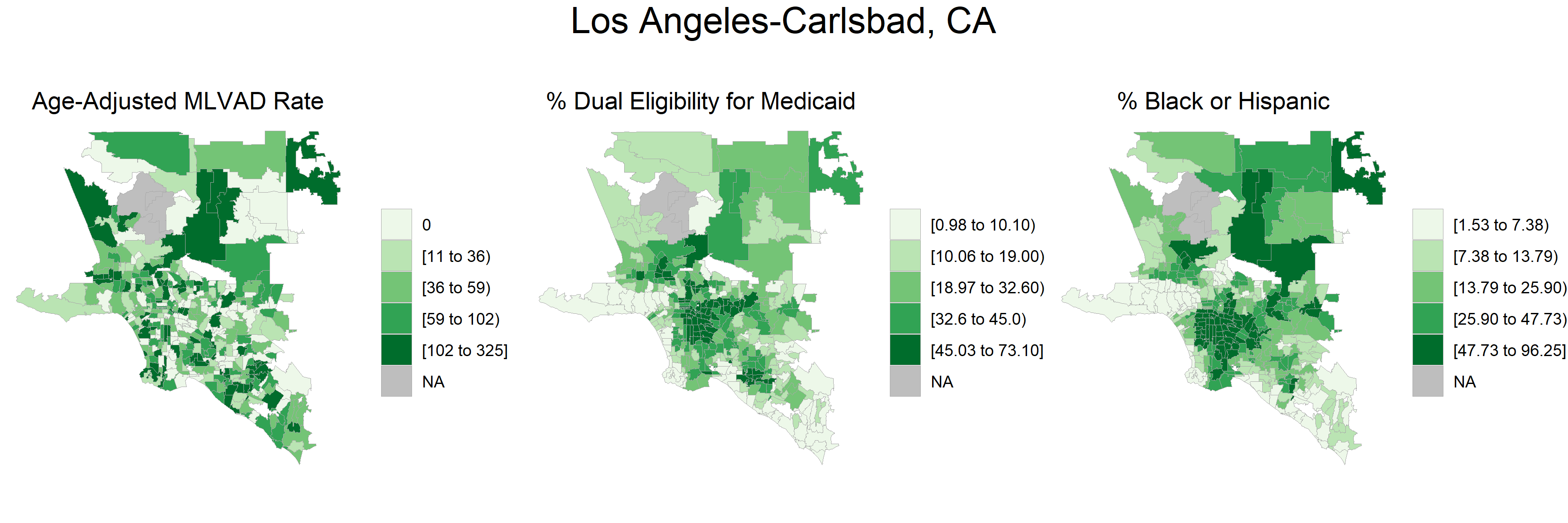

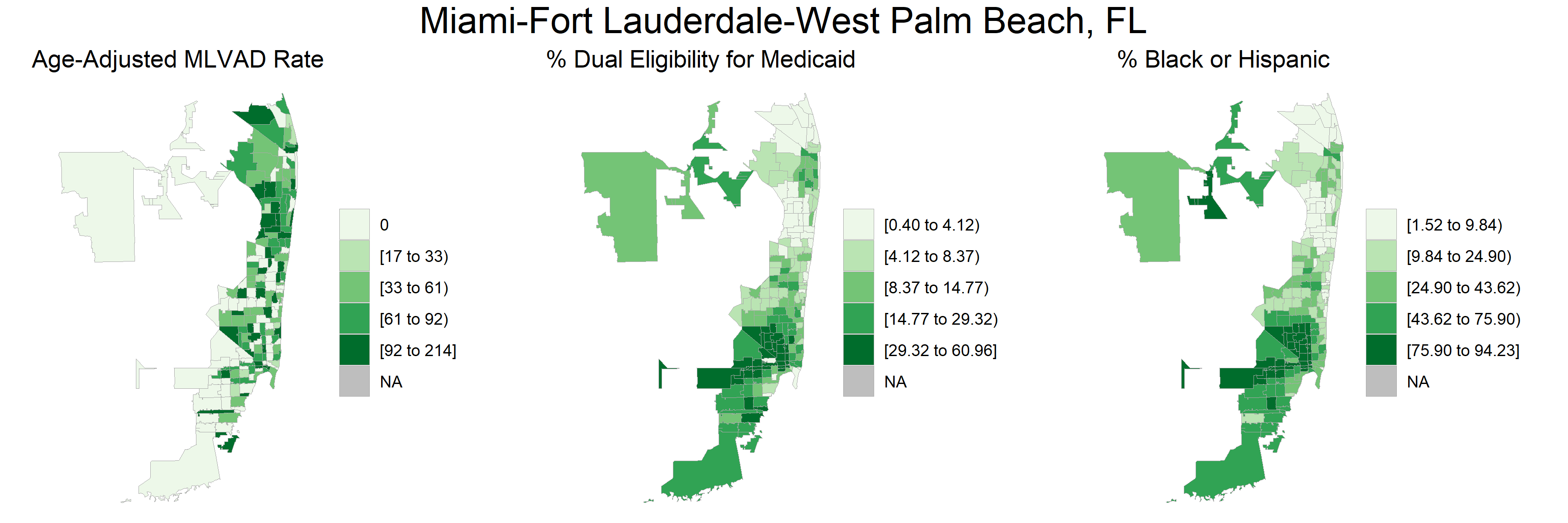

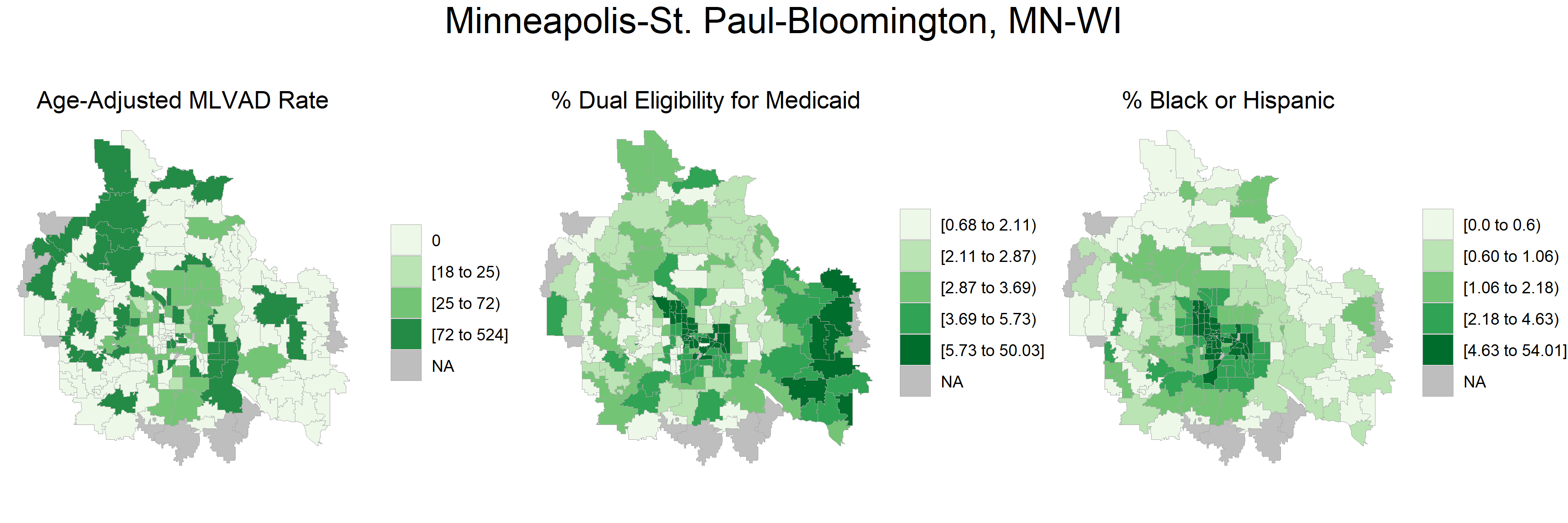

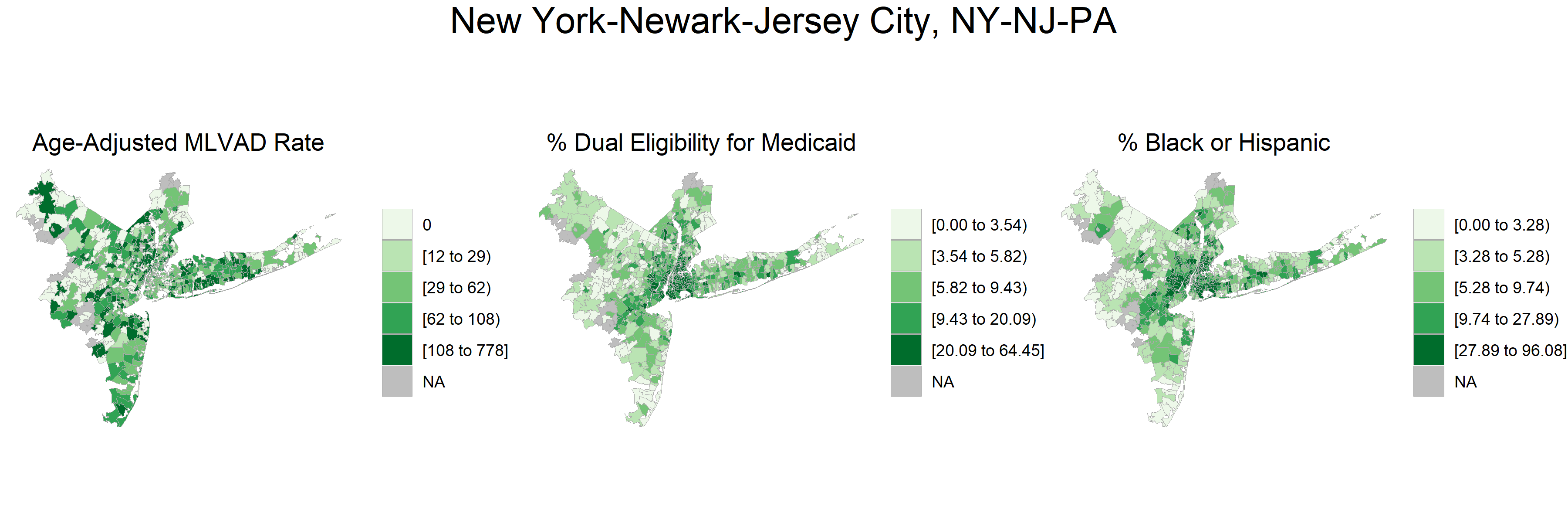

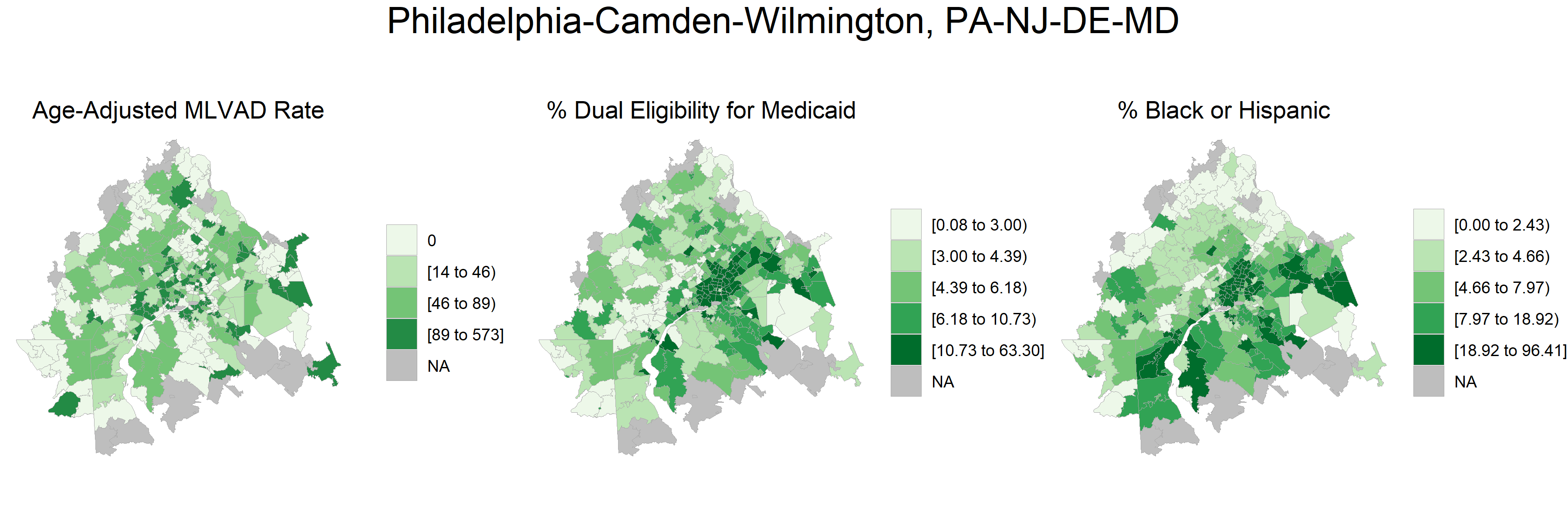

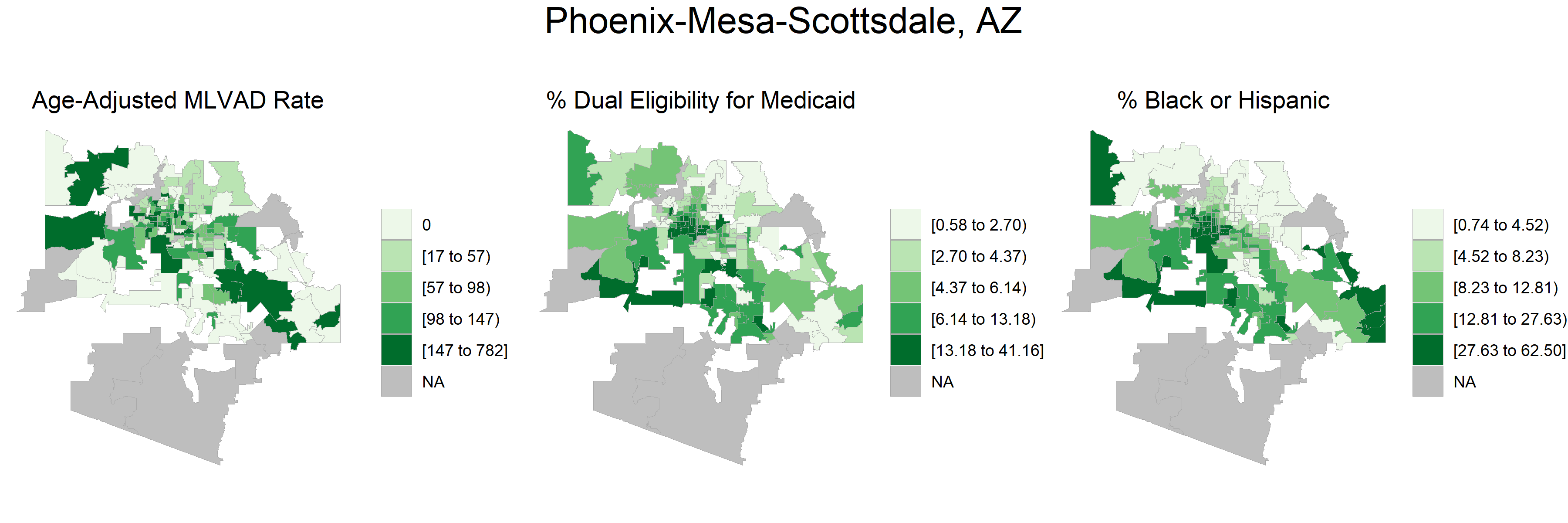

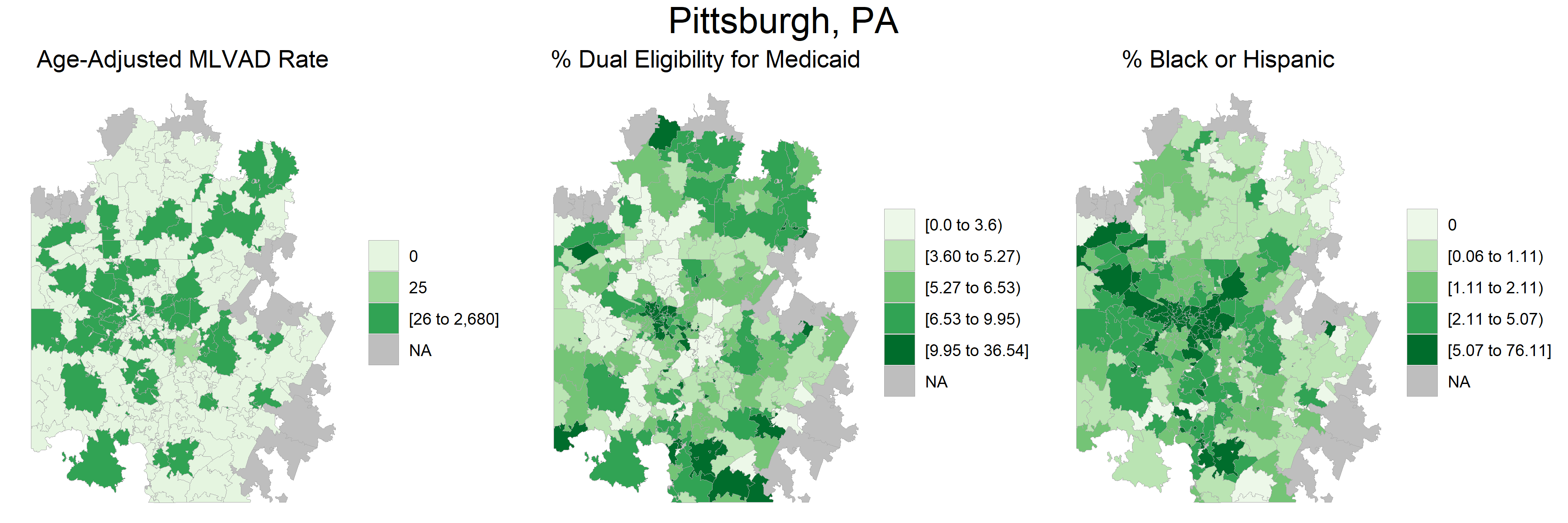

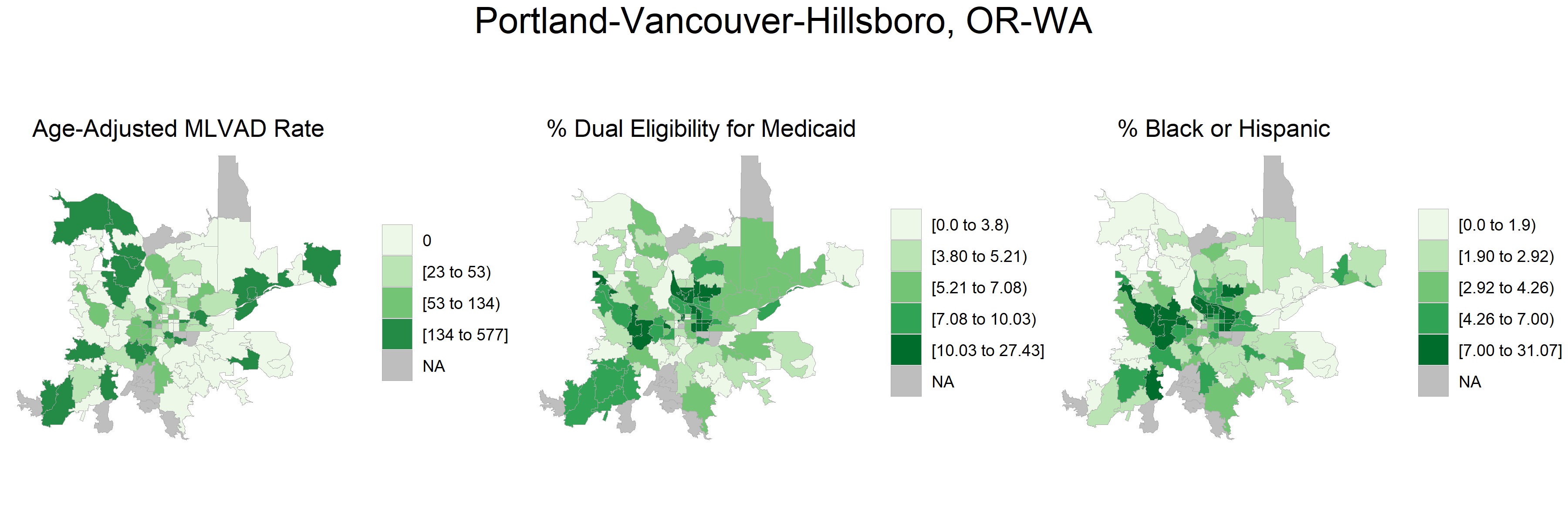

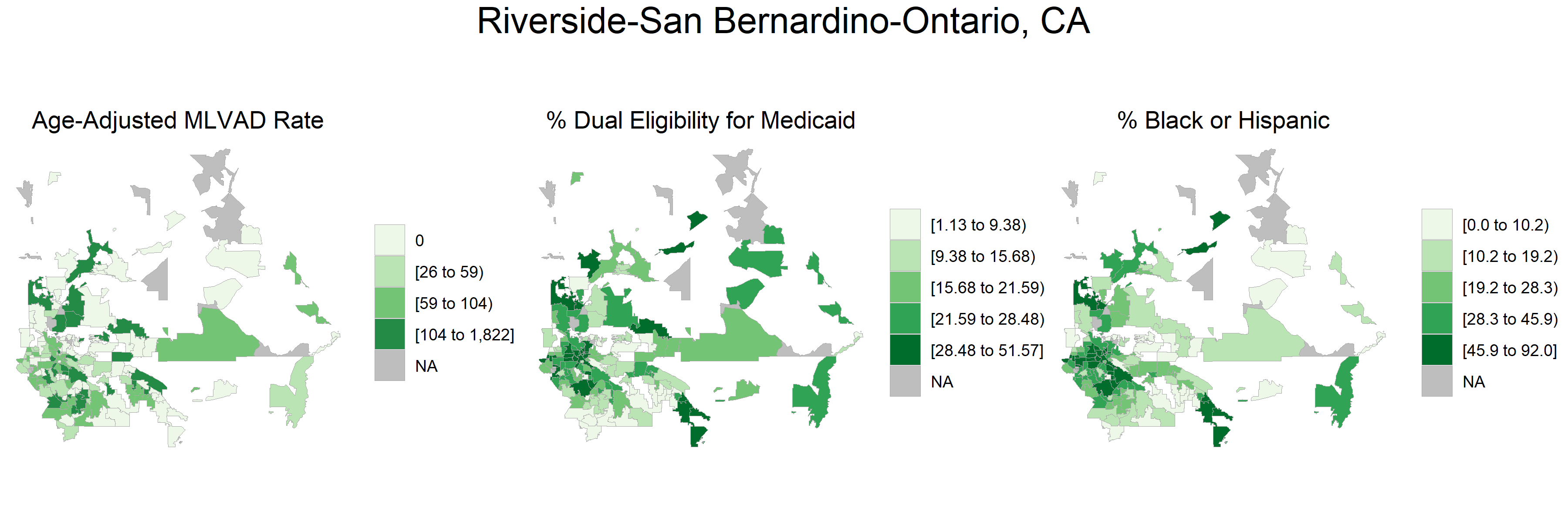

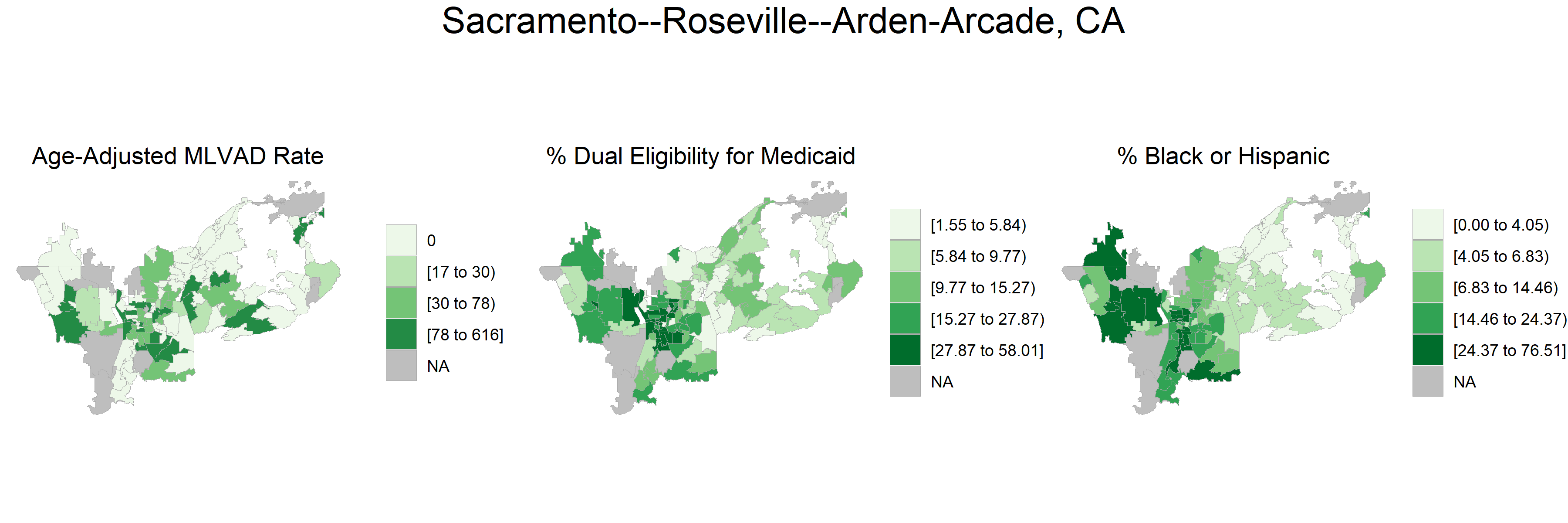

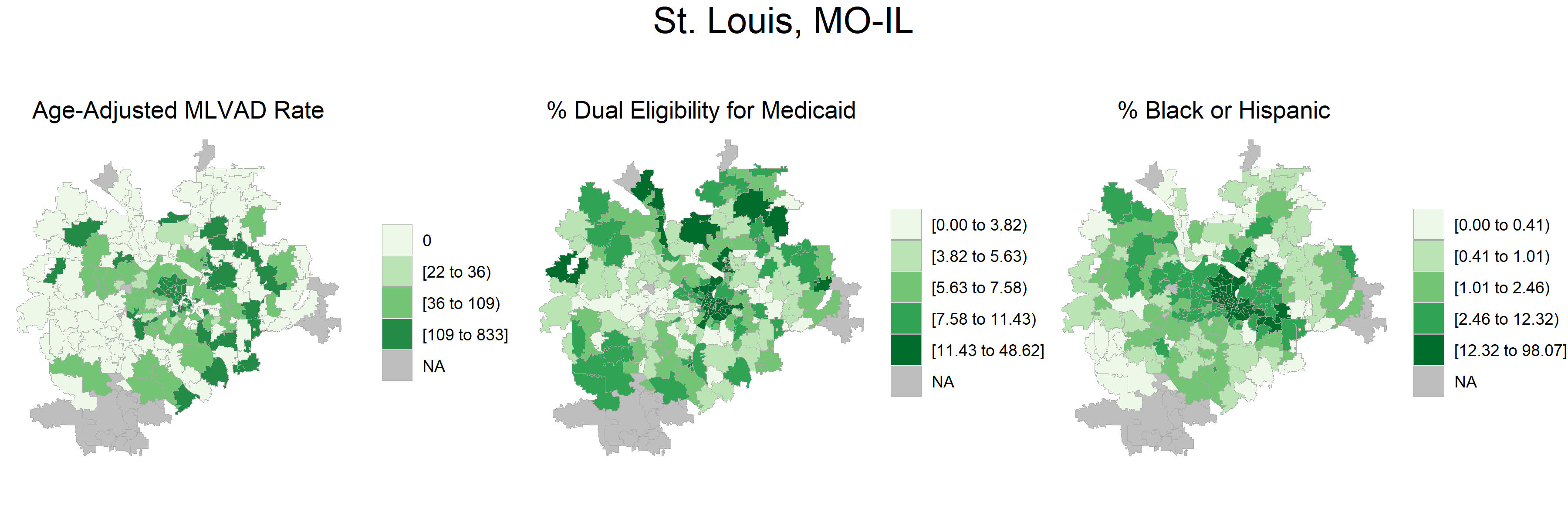

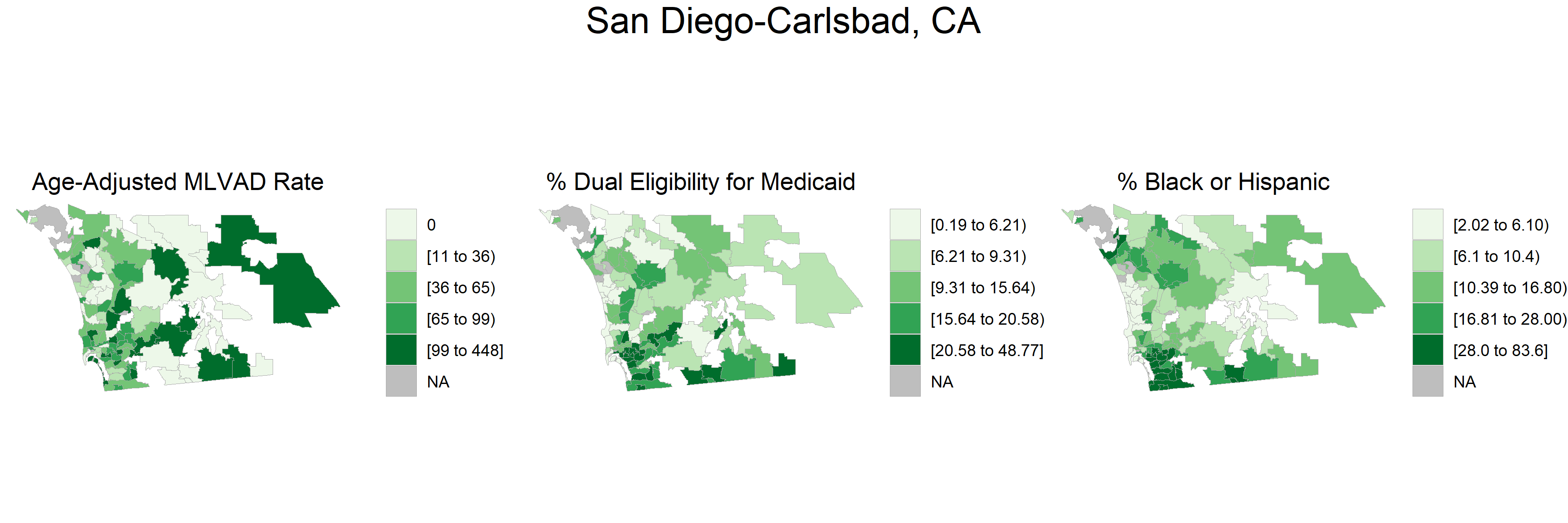

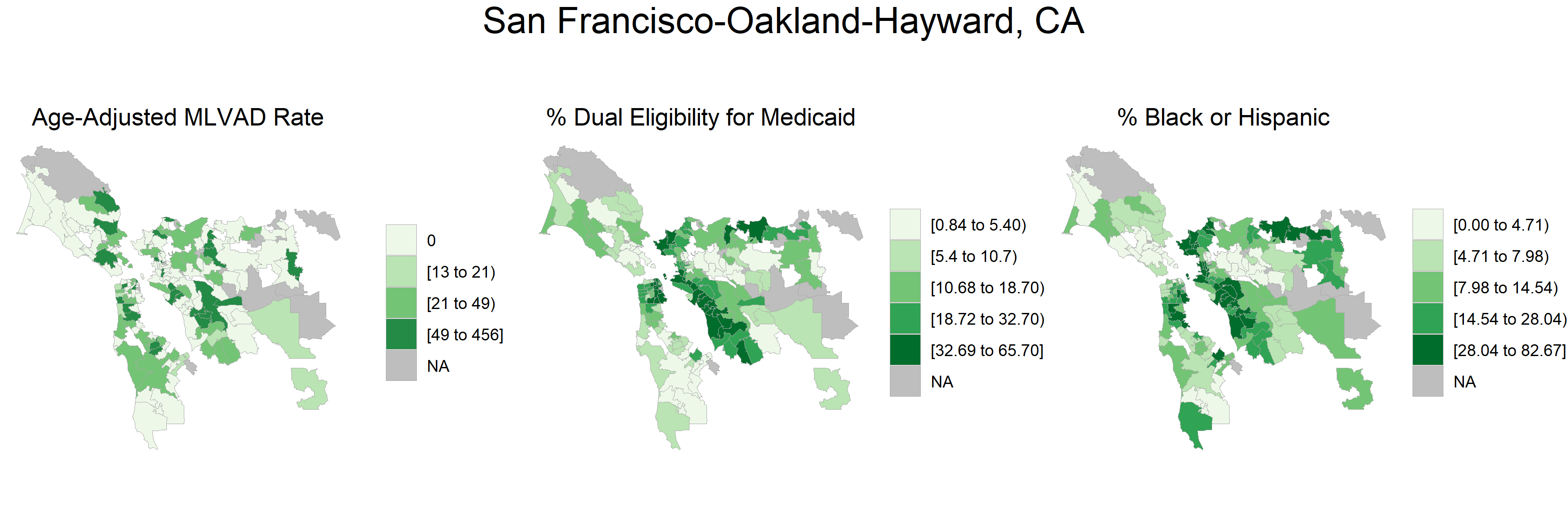

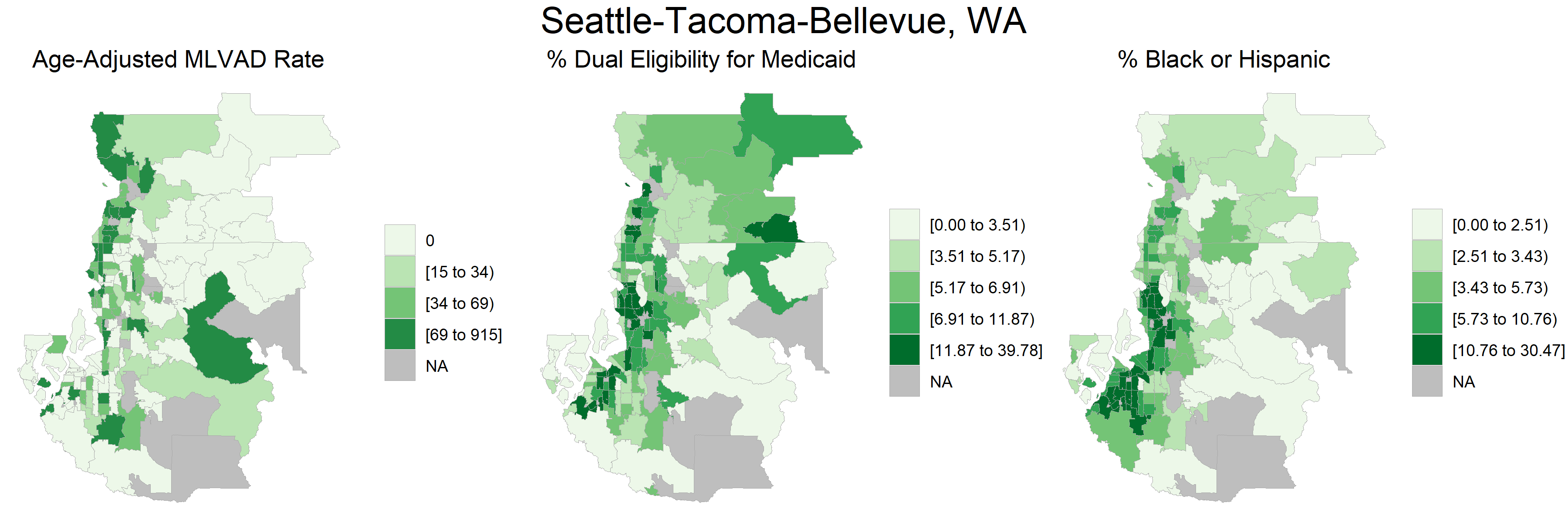

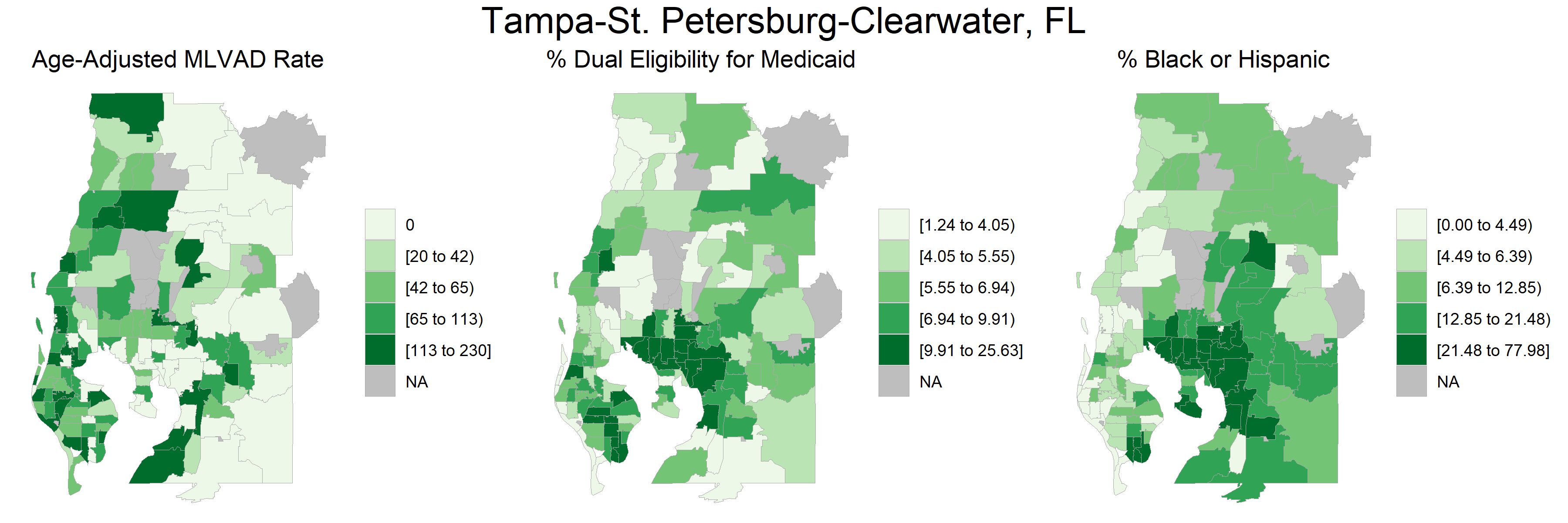

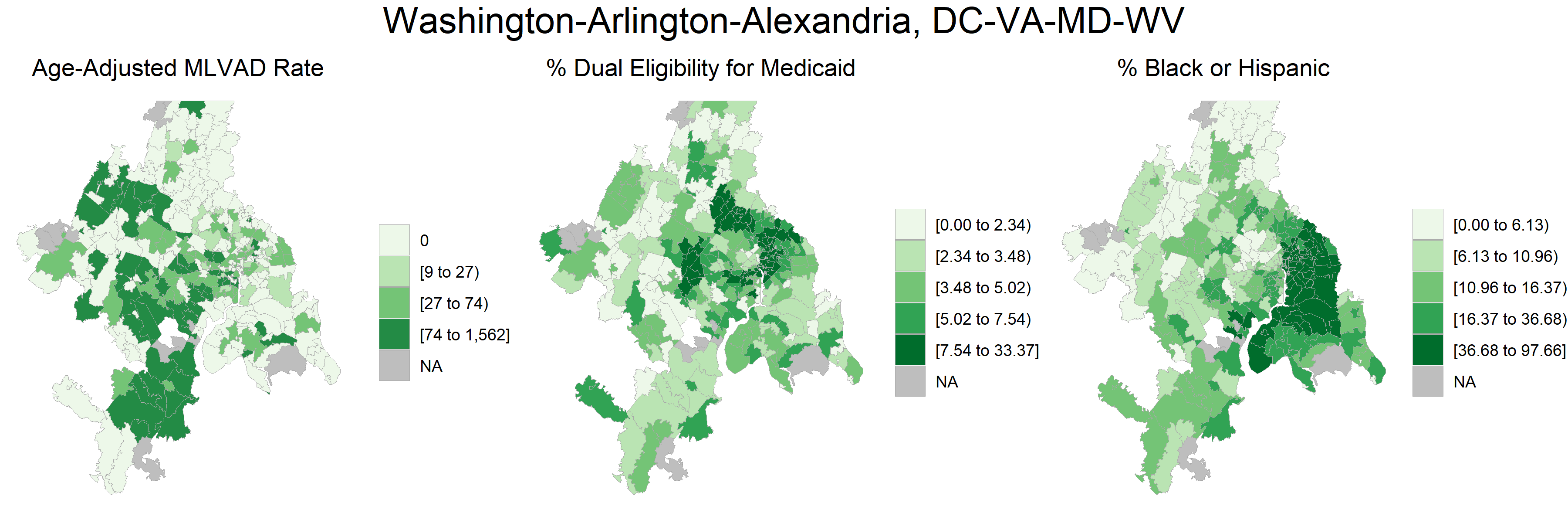


**Supplemental Figure 3**. Age-adjusted mLVAD/ECMO Rates among all Medicare beneficiaries, % Dual Eligibility for Medicaid, and % Black or Hispanic patients in ZIP Codes in 25 largest CBSAs with mLVAD/ECMO programs.


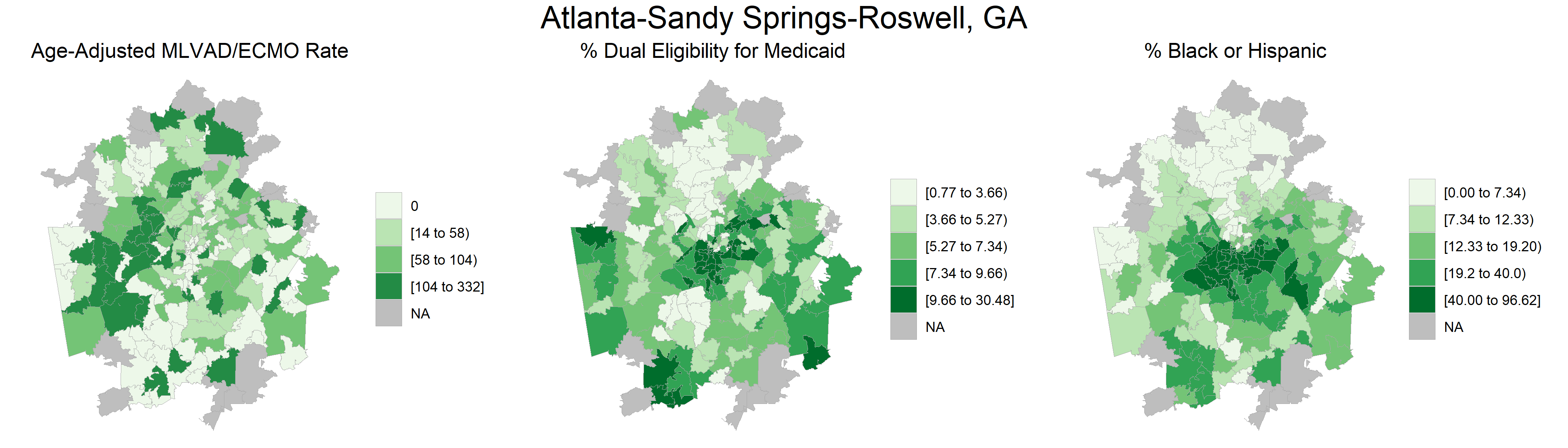

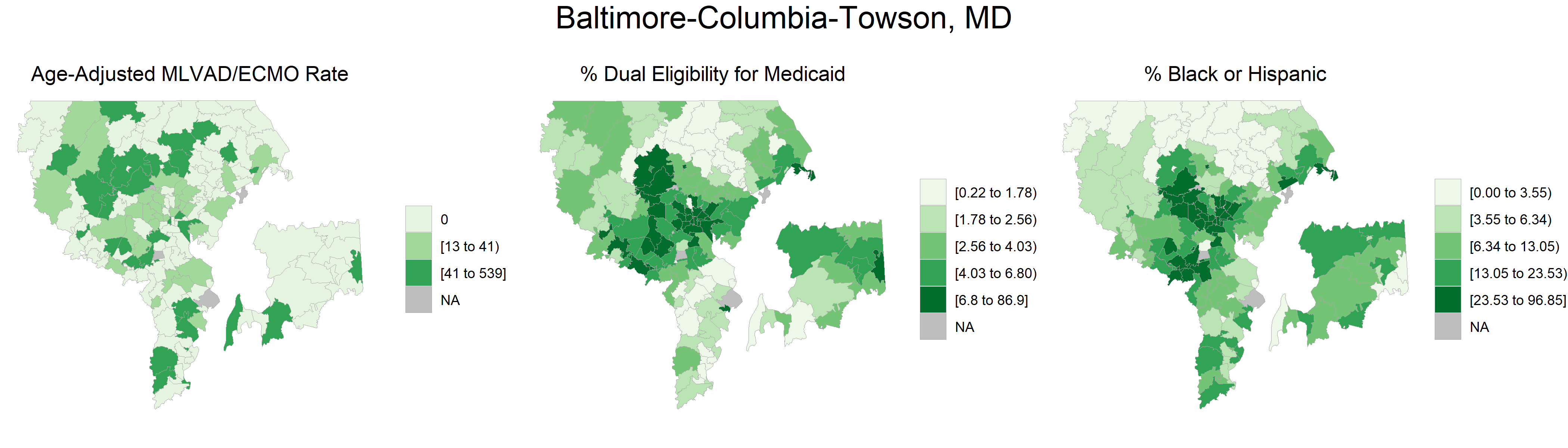

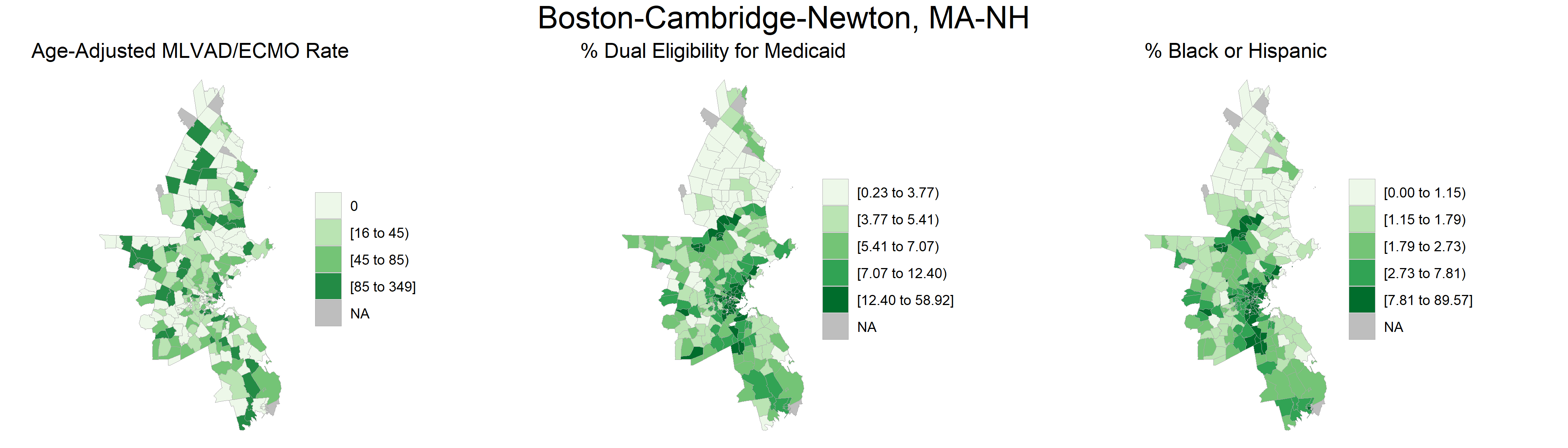

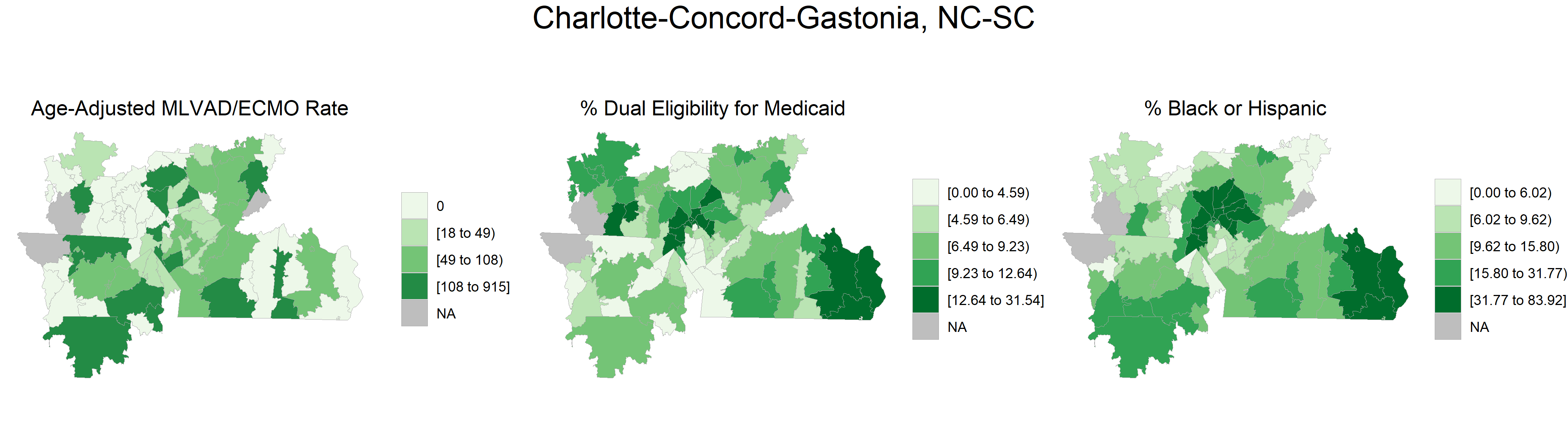

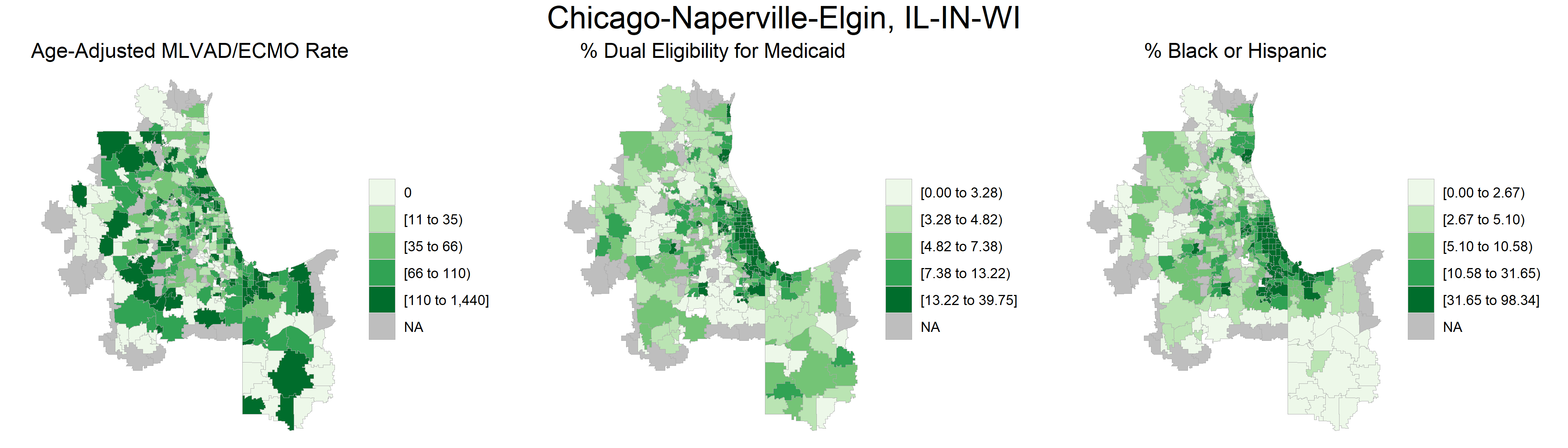

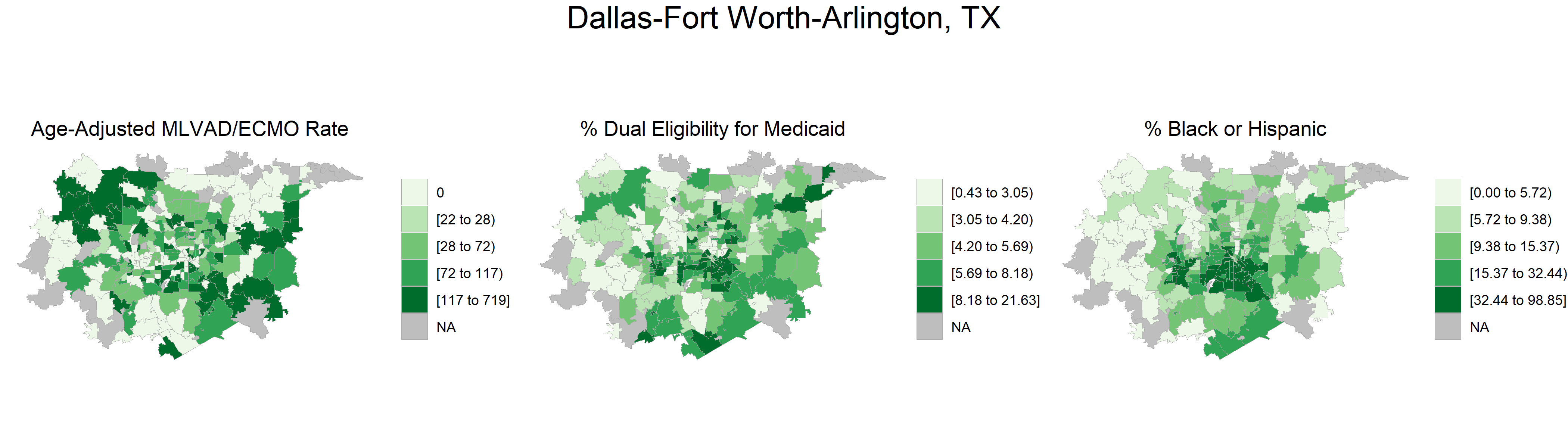

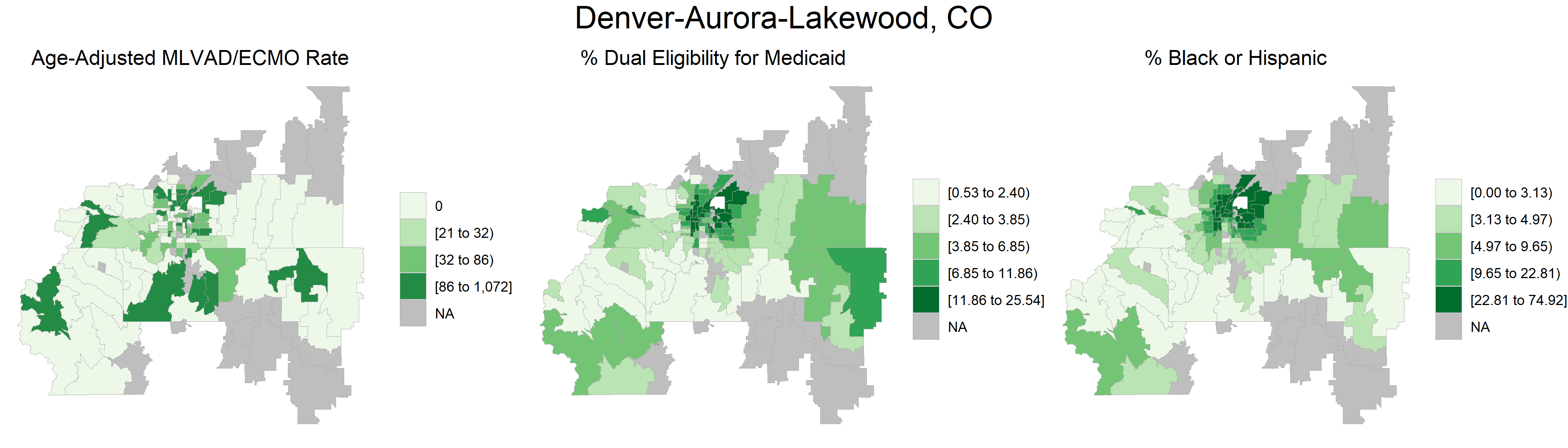

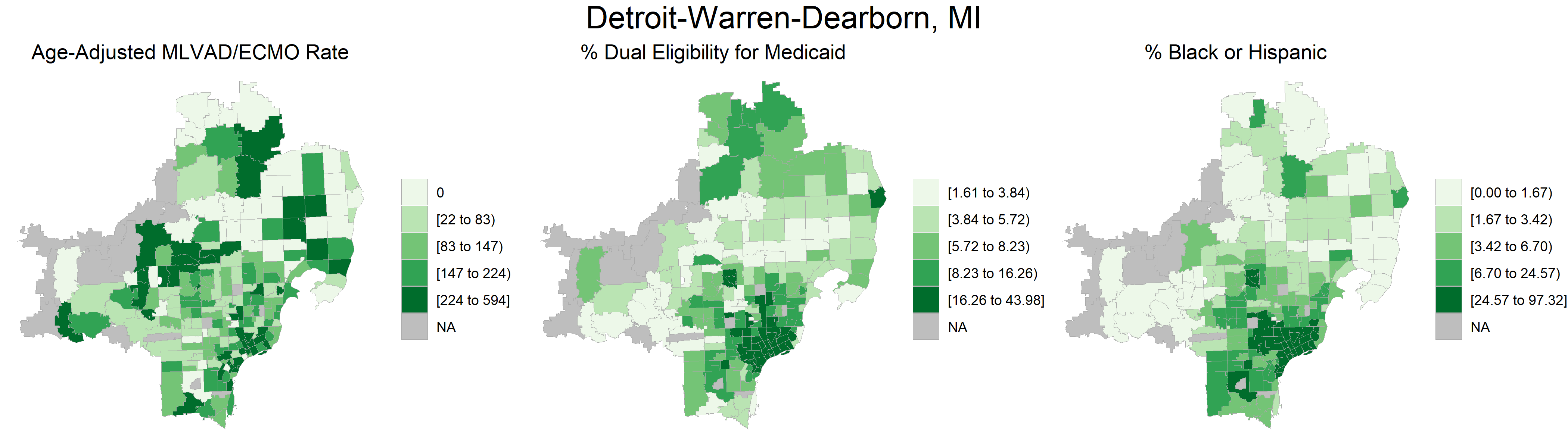

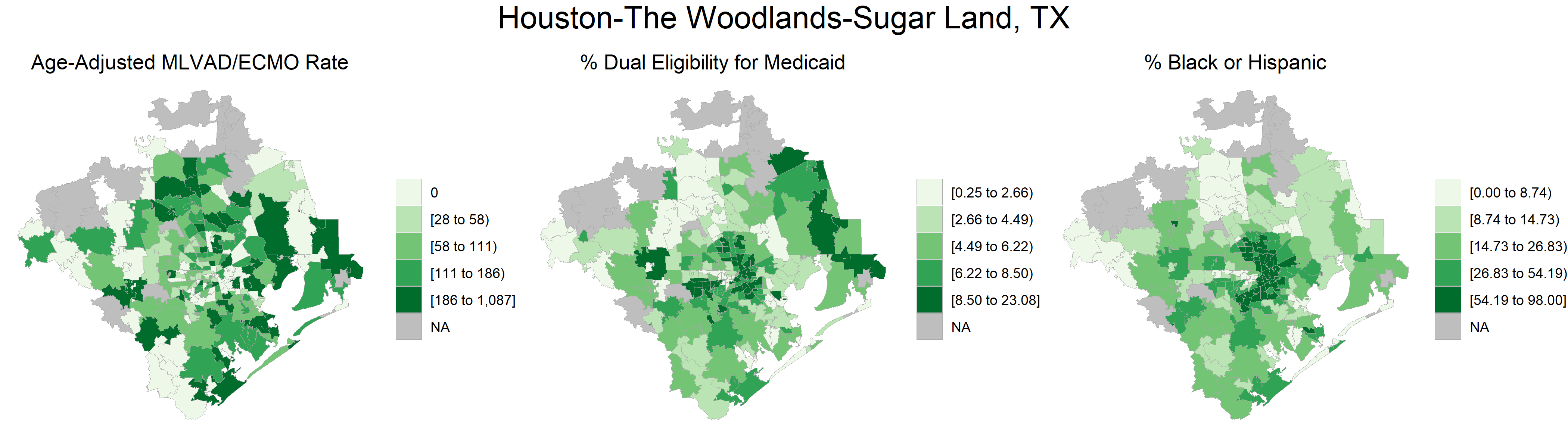

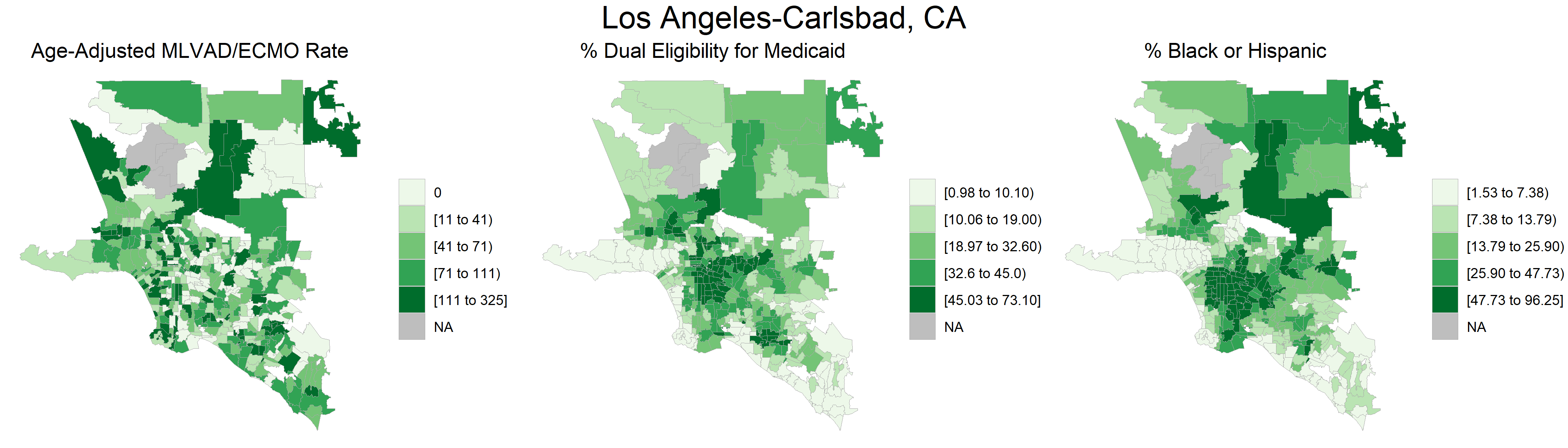

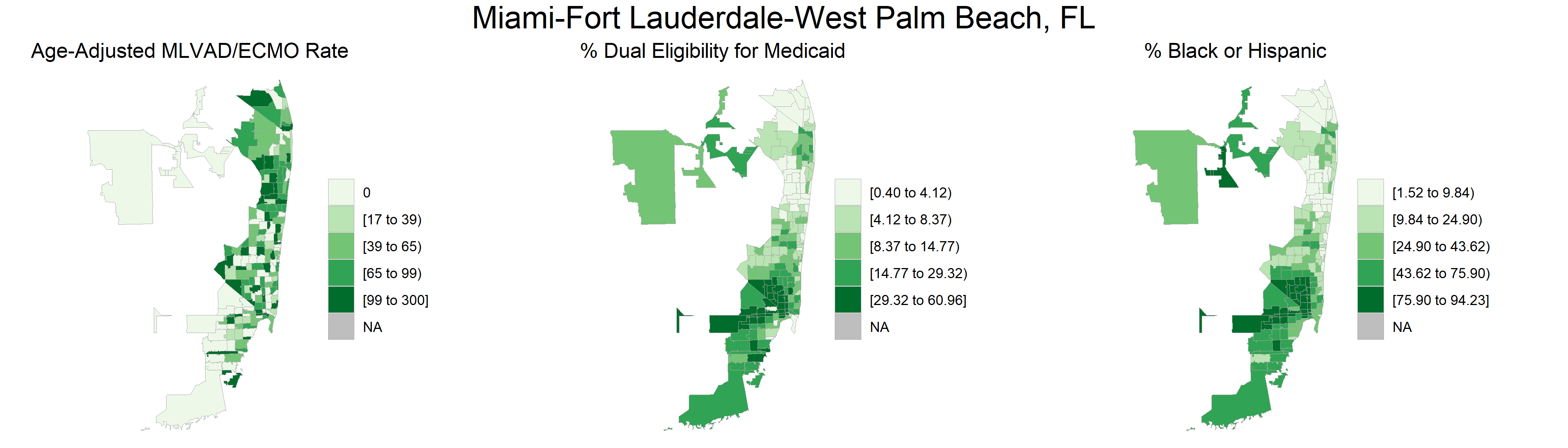

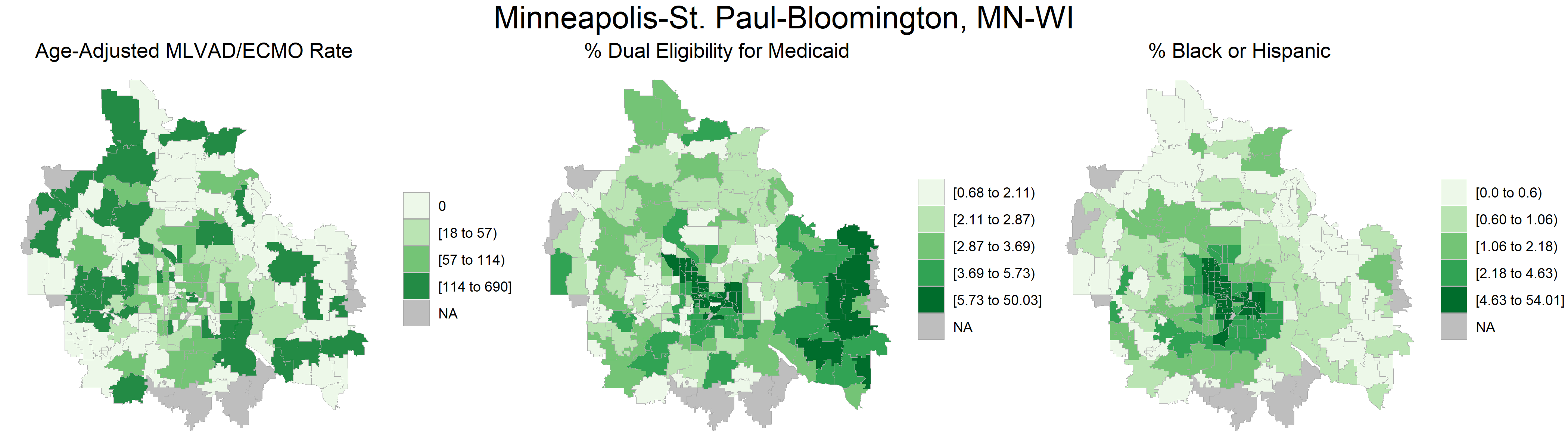

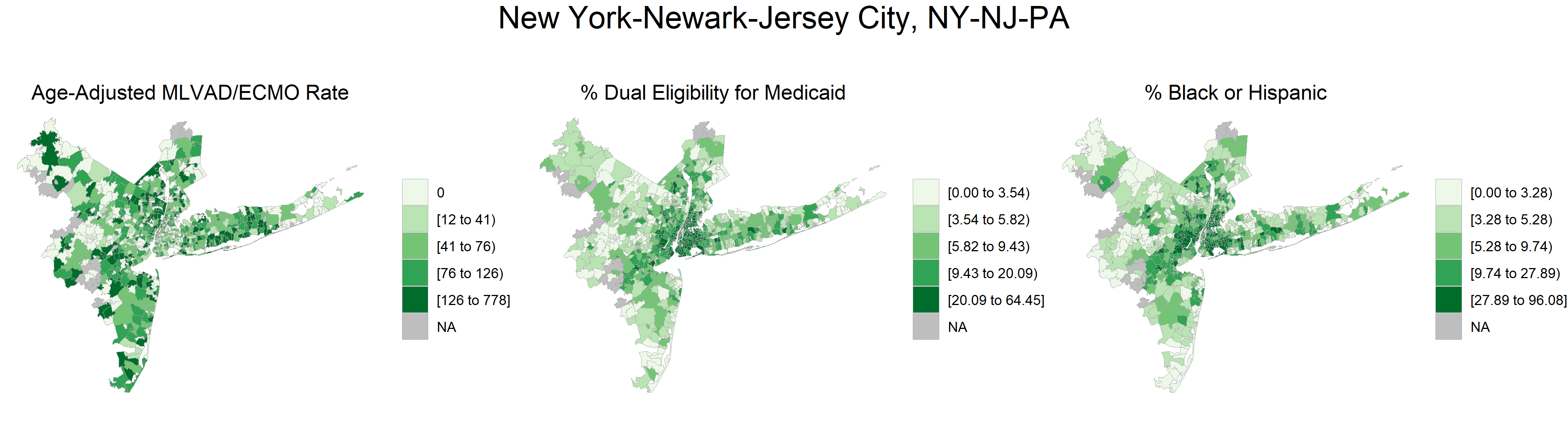

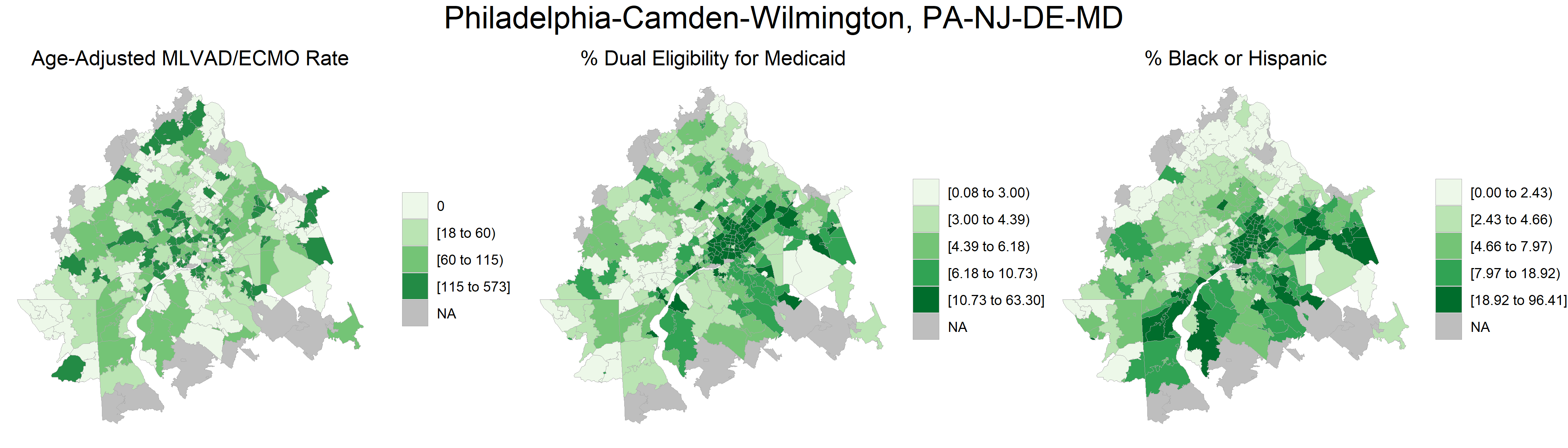

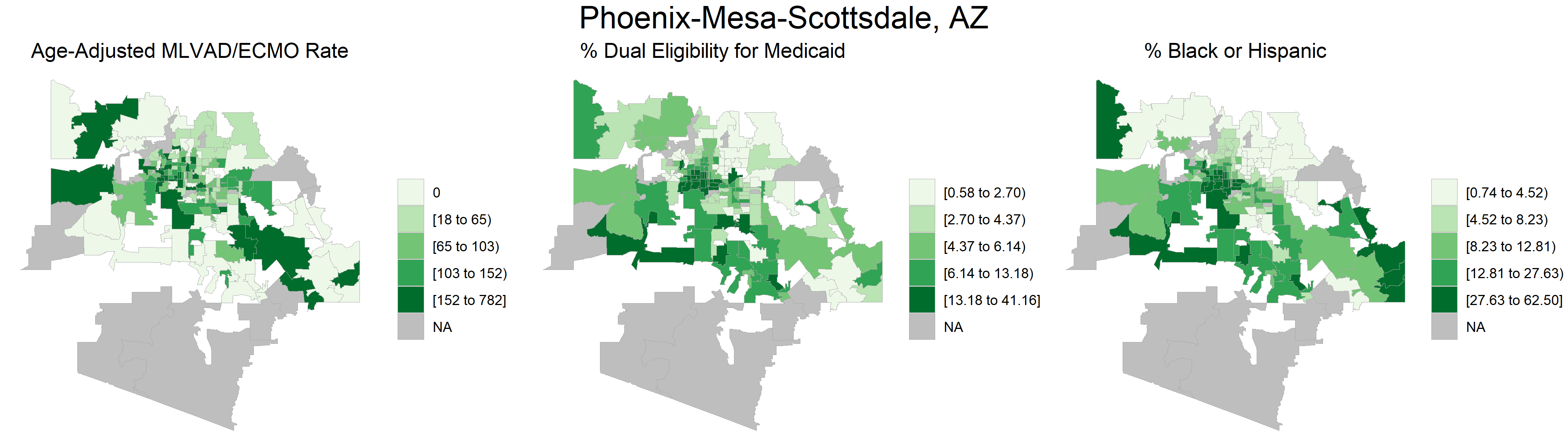

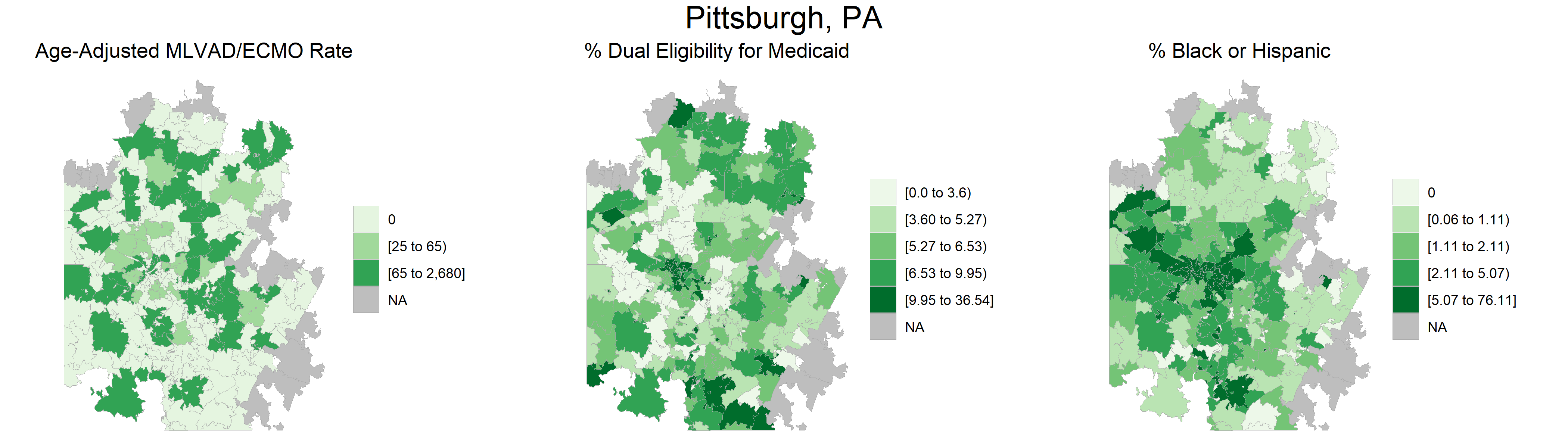

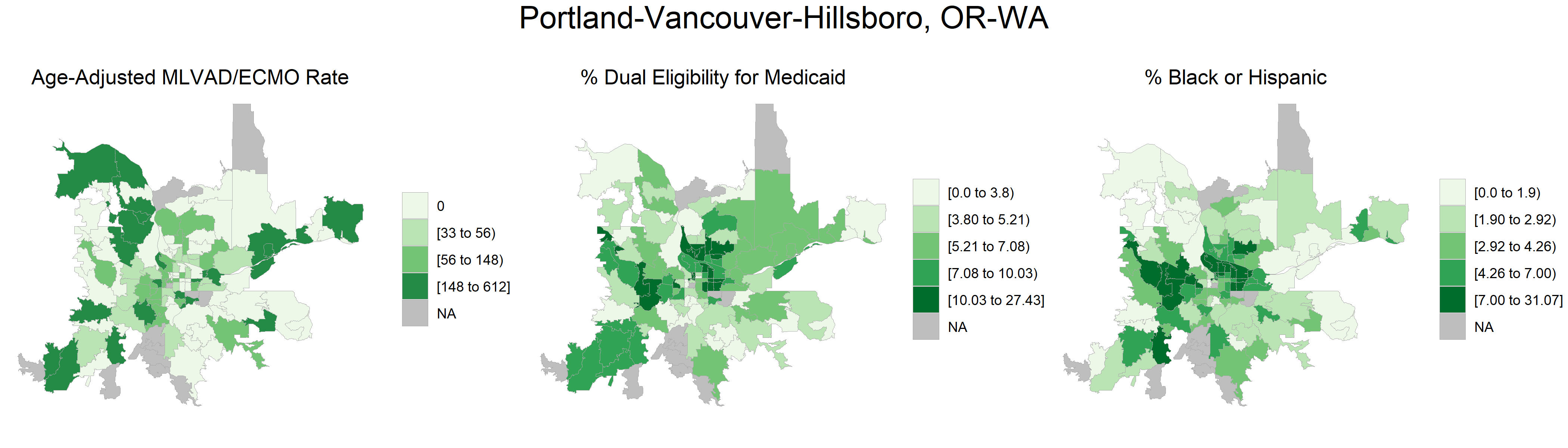

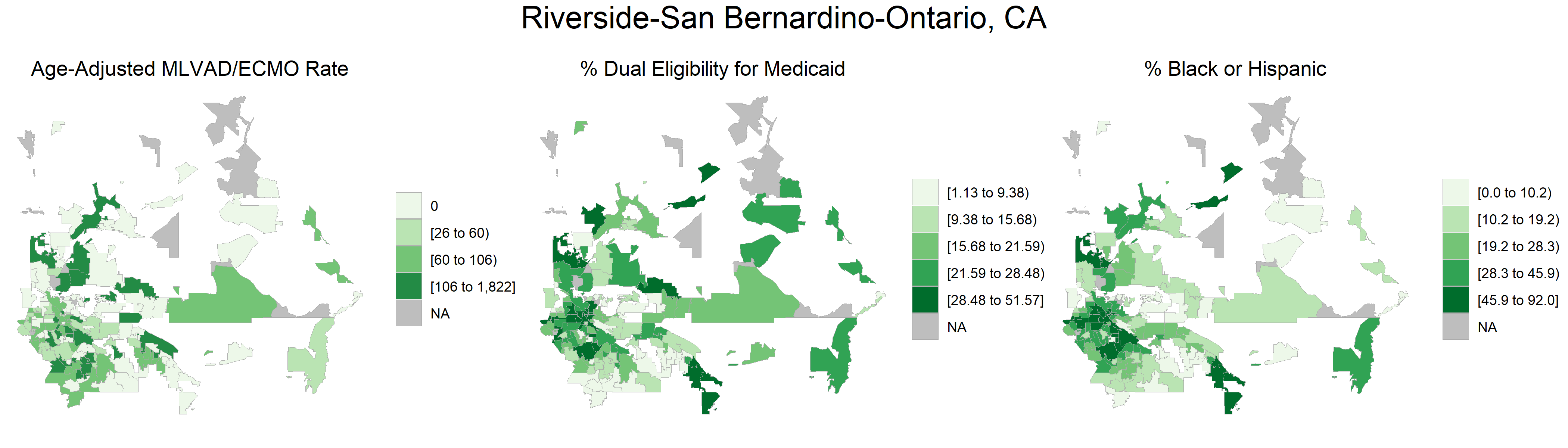

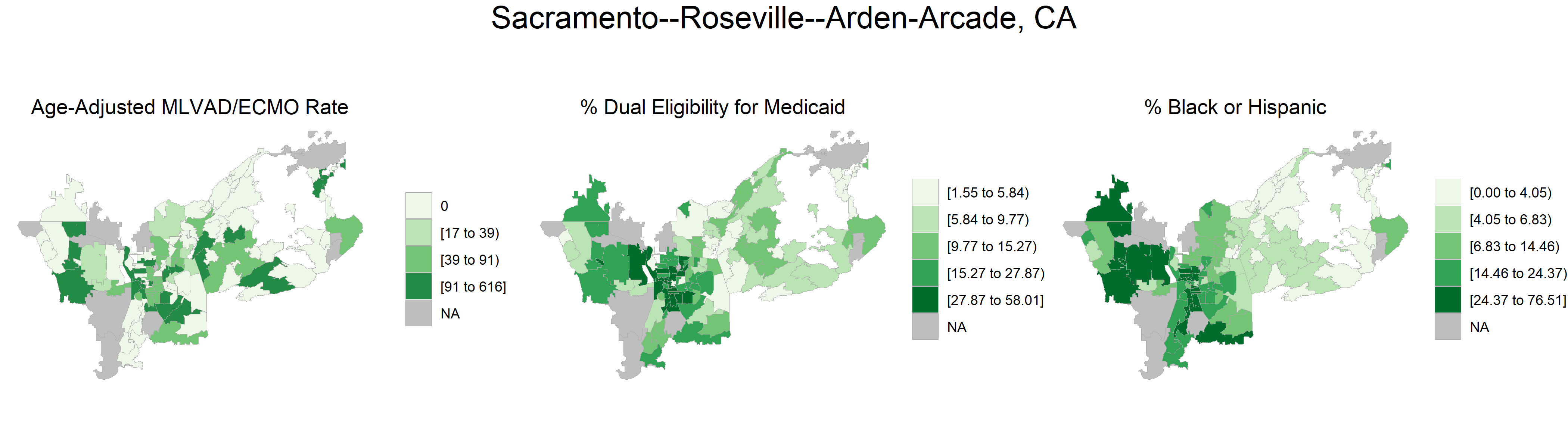

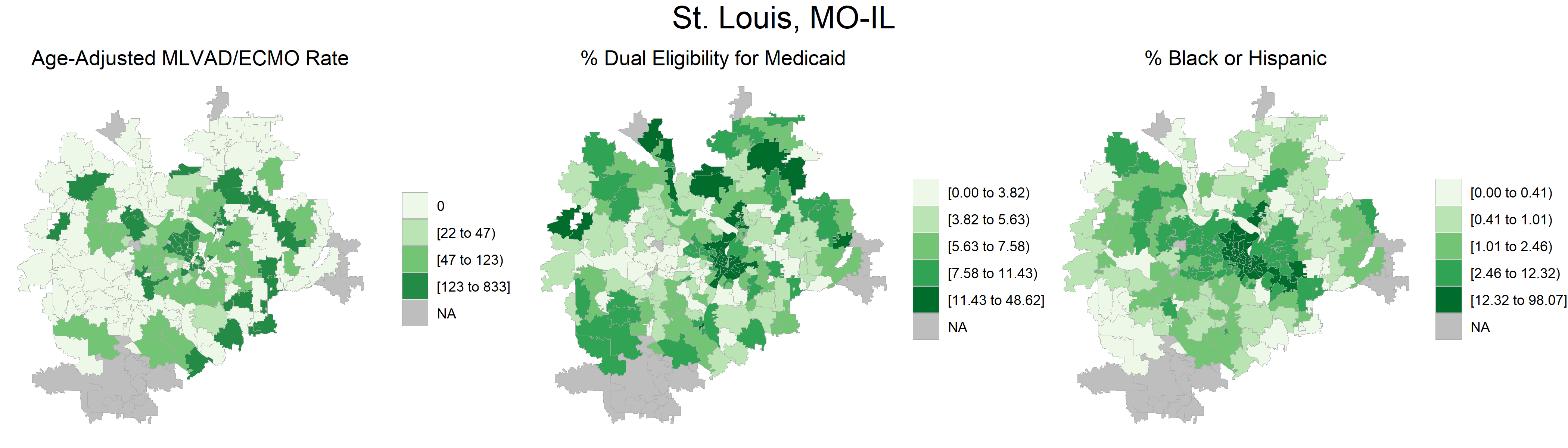

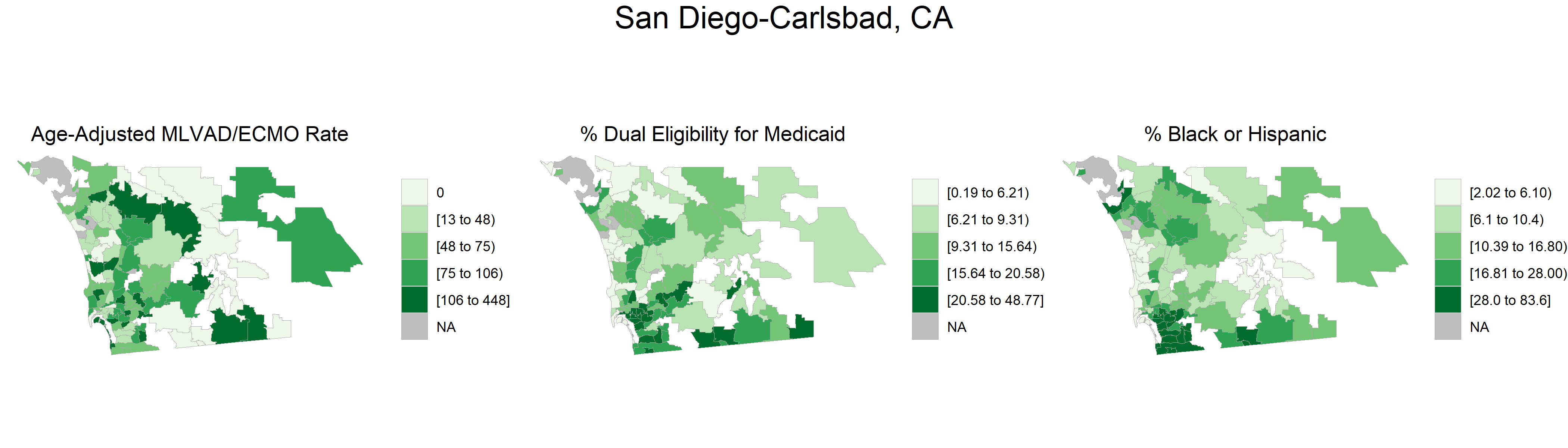

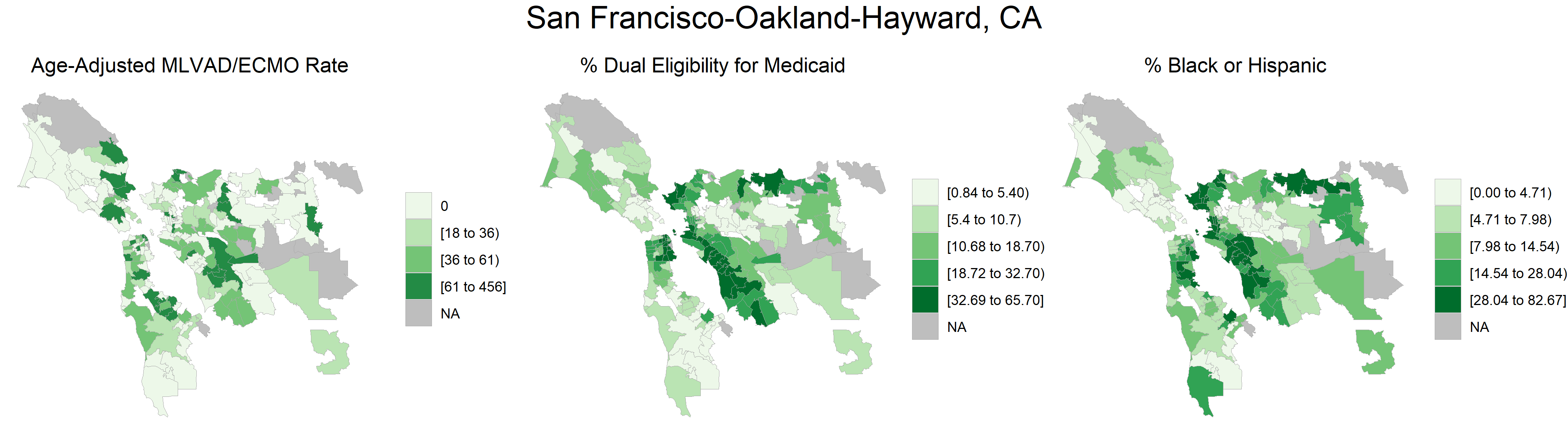

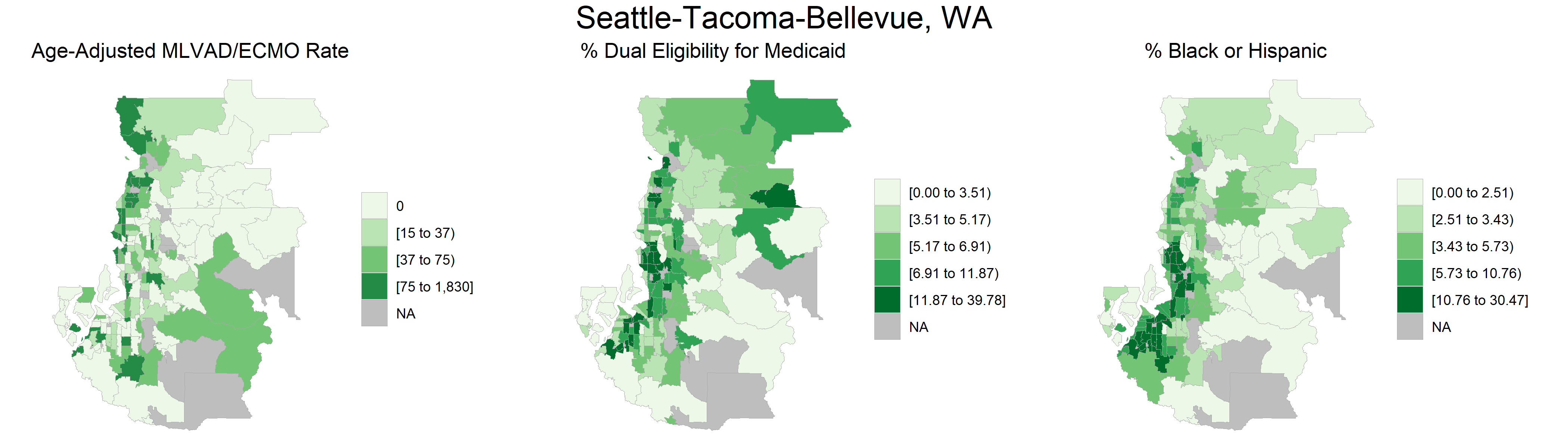

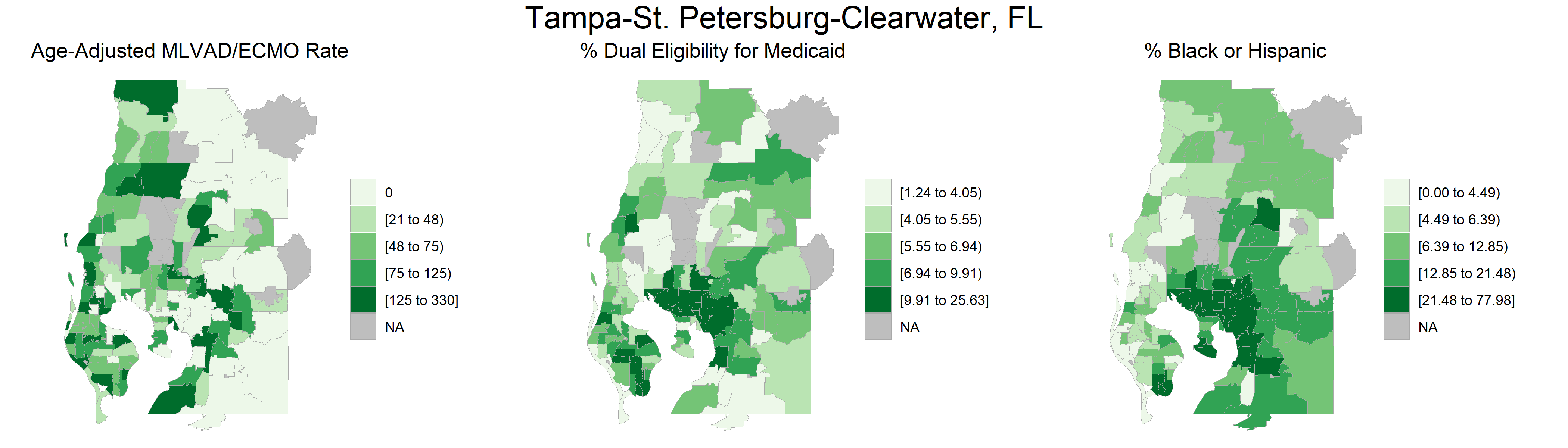

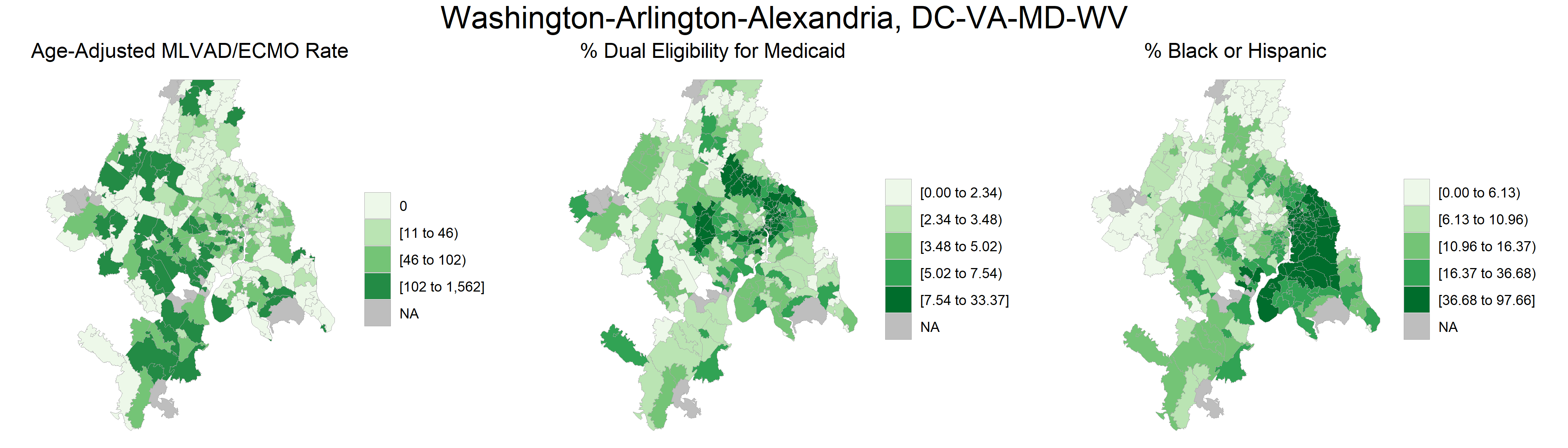

Supplement: Supplemental Figures S1-S3 and Supplemental Tables S1-S5 [file mmc1.docx]
